# Tolerability profile of paliperidone palmitate formulations: A pharmacovigilance analysis of the EUDRAVigilance database

**DOI:** 10.3389/fpsyt.2023.1130636

**Published:** 2023-04-06

**Authors:** Giuseppe Cicala, Renato de Filippis, Maria Antonietta Barbieri, Paola Maria Cutroneo, Pasquale De Fazio, Georgios Schoretsanitis, Edoardo Spina

**Affiliations:** ^1^Department of Clinical and Experimental Medicine, University of Messina, Messina, Italy; ^2^Psychiatry Unit, Department of Health Sciences, University Magna Graecia of Catanzaro, Catanzaro, Italy; ^3^Sicilian Regional Pharmacovigilance Center, Azienda Ospedaliera Universitaria Policlinico G. Martino, Messina, Italy; ^4^Department of Psychiatry, Psychotherapy and Psychosomatics, Hospital of Psychiatry, University of Zurich, Zurich, Switzerland; ^5^The Zucker Hillside Hospital, Department of Psychiatry Research, Northwell Health, Glen Oaks, NY, United States; ^6^Department of Psychiatry, Zucker School of Medicine at Northwell/Hofstra, Hempstead, NY, United States

**Keywords:** adverse drug reaction, antipsychotics, schizophrenia, pharmacovigilance, paliperidone palmitate, long-acting injectable, drug-induced reaction, EUDRAVigliance

## Abstract

**Introduction:**

Long-acting injectable antipsychotics (LAIs) have proven to be effective in the maintenance treatment of patients suffering from schizophrenia, and their safety and tolerability profiles represent a key factor in their long-term use and choice in clinical practice. Paliperidone palmitate (PP) is the only second-generation LAI (SGA-LAI), available in both one- (PP1M) and 3-month (PP3M) formulations. However, real-world prospective studies on PP1M and PP3M are still few and mostly conducted on small samples. In this context, we aimed to better define the safety and tolerability profile of PP using real world pharmacovigilance data.

**Methods:**

We retrospectively analyzed the publicly available data regarding Individual Case Safety Reports (ICSRs), presenting PP1M and/or PP3M as suspected drugs, reported on EUDRAVigilance between 2011 and June 30th, 2022. ICSRs relative to at least one SGA-LAI other than PP, reported between 2003 and June 30th, 2022, were also examined as reference group. Data were evaluated with a descriptive analysis, and then, as disproportionality measures, crude reporting odds ratio (ROR) and 95% confidence interval (CI) were calculated.

**Results:**

A total of 8,152 ICSRs met the inclusion criteria, of those 77.7% (*n* = 6,332) presented as suspected drug PP1M, 21.2% (*n* = 1,731) PP3M, while 89 cases indicated both PP1M and PP3M. Significantly higher probabilities of reporting in PP-related reports were observed for the primary Standardized MedDRA Queries “Sexual Dysfunctions” (ROR = 1.45; 95% CI 1.23-1.70), “Haemodynamic oedema, effusions and fluid overload” (ROR = 1.42; 1.18-1.70), as well as “Fertility disorders” (ROR = 2.69; 1.51-4.80).

**Discussion:**

Our analysis indicates that the tolerability and safety profiles of PP are in line with what is known for the other SGA-LAIs. However, differences regarding endocrine system ADRs have been noticed. The results presented in this work do not discourage the prescription of SGA-LAI formulations but aim to enhance their safety.

## 1. Introduction

Antipsychotic medications represent the mainstay of the pharmacological treatment of schizophrenia (SCZ) (1). They are commonly categorized into three drug classes, first- (FGAs), second- (SGAs) and third-generation antipsychotics (TGAs) (2). Poor adherence to antipsychotic treatment is a critical aspect of the clinical management of patients affected by schizophrenia spectrum disorders (SSDs). In addition, treatment discontinuation represents a relevant risk factor for relapse and rehospitalization (3–6). To improve antipsychotic adherence in patients affected by SCZ in the 1960s the long-acting injectable (LAI) antipsychotic formulations, initially based on FGAs (FGA-LAIs), were introduced (7, 8).

The LAI formulations have proven to reduce the risk of relapse and re-hospitalization due to non-adherence (9). This makes them valuable therapeutic options for the long-term management of patients suffering from SSDs (10–13). Furthermore, robust literature evidence suggests that LAIs may also provide an effective treatment strategy for patients in the early-phase or with a first-episode of psychosis (FEP) (12, 14–16).

As for their oral counterparts, FGA-LAIs have been gradually less prescribed due to the risk of extrapyramidal symptoms and tardive dyskinesia (17, 18). However, over the past 20 years, several SGAs, including olanzapine, risperidone, and paliperidone, and one TGA, aripiprazole, have become available, partially replacing FGA-LAIs thanks to a lower liability for movement disorders (19). There are considerable differences between second-generation LAIs (SGA-LAIs) regarding pharmacodynamic and pharmacokinetic profiles, injection interval, cost, requirements for oral supplementation, and risks of adverse events (20, 21). Safety profiles of SGA-LAIs generally follow the known profiles of the oral molecule, although unexpected safety signals were occasionally observed in clinical practice (22).

Among the currently available SGA-LAIs, paliperidone palmitate (PP) (the esterified form of paliperidone, an active metabolite of risperidone) is the only one already available in both a monthly (PP1M) and a quarterly formulation (PP3M), with a recently approved 6-month PP (PP6M) formulation (23). In particular, the PP3M formulation has shown significant efficacy in delaying the time to relapse in patients suffering from SCZ (24, 25). Candidates for PP3M are patients previously prescribed PP1M (26). In other words, patients introduced to PP3M have been previously exposed PP1M, which they may tolerate well before clinicians switch them to PP3M. This could be related to the low incidence of adverse drug reactions (ADRs) (27).

The aim of the present study was to analyze the ADRs related to PP1M and PP3M and to compare them to those related to the other SGA-LAIs, in the Spontaneous Reporting System (SRS) database (i.e., European Union Drug Regulating Authorities Vigilance database; EUDRAVigilance) of the European Medicines Agency (EMA).

## 2. Materials and methods

### 2.1. Data source

Data on Individual Case Safety Reports (ICSRs) presenting as suspected drugs LAI formulations of PP (i.e., PP1M and/or PP3M) were retrieved using the EUDRAVigilance access platform (publicly available at www.adrreports.eu). EUDRAVigilance functions as a management and analysis platform for information on suspected ADRs regarding drugs that have obtained marketing authorization or are currently under evaluation in clinical trials across the European Economic Area (EEA). More specifically, EUDRAVigilance represents the collection point for all the ICSRs (regarding either drugs or vaccines), reported by healthcare professionals (HCPs) and non-HCP figures to any of the European Union (EU) competent authorities at the national level or the marketing authorization holder. The EU medicines regulatory network, in the form of the EMA, acts as the responsible authority for the maintenance and constant update of EUDRAVigilance. For transparency’s sake, data collected on EUDRAVigilance are publicly available through the previously cited access portal. Data are made available in different tiers of completeness, with the more specific ones requiring access authorization directly licensed from the EMA. The data access level used for the analysis was the one indicated as “Stakeholder Group II: Healthcare professionals, patients and the general public” in the EUDRAVigilance access policy (28).

### 2.2. Selection of individual case safety reports

All ICSRs reported as suspected drugs LAI formulations of PP were retrieved using the line-listing function of the EUDRAVigilance platform. The timeframe used for report collection spanned between January 1st, 2011 (the year of the first market approval for PP1M) and June 30th, 2022. The reference Group (RG) for the analyses was constituted by ICSRs showing at least one SGA-LAI other than PP (i.e., LAI formulations of aripiprazole, olanzapine, and risperidone) as suspected drugs reported to EUDRAVigilance between January 1st, 2003, the year of commercialization of risperidone LAI, and June 30th, 2021. The authors acknowledge that aripiprazole belongs to the class of TGAs (2). However, concerning LAI formulations, a number of literature sources enlist aripiprazole-based LAIs as part of SGA-LAIs (29, 30). Thus, after careful consideration, to improve the applicability of the analysis results, aripiprazole LAI-related ICSRs were considered in the reference group. The retrieved dataset included the following fields: ICSR identification number in EUDRAVigilance; date of receipt; primary source qualification; the presence of an eventual literature reference; patients’ sex and age group; ADRs characteristics (type of ADR, duration, outcome, and seriousness status) and characteristics of suspected and concomitant drugs (Type of drug, use indication, duration of therapy, drug dose and administration route). The level of data completion varied for each ICSR. Once retrieved, ICSRs identified as “non-spontaneous,” ICSRs linked to literature sources, and ICSRs that presented as suspected drugs vaccines have all been excluded.

### 2.3. Data analysis

Data regarding the available demographic characteristics of patients (i.e., sex and age group) were evaluated by means of a descriptive analysis. The descriptive analysis also included adverse event characteristics (i.e., outcome and seriousness), primary source qualification, and the number of suspected drugs other than the LAI formulations of PP. The latter is described cumulatively for all PP-related and for each PP formulation. In addition to that, the annual trend in ICSRs reporting was also evaluated. All the ADRs were classified in accordance with the Medical Dictionary for Regulatory Activities (MedDRA^®^), which follows a hierarchical structure in which terms are organized into five levels. Observations are codified, at first with more specific lowest-level terms (LLT), to resemble the clinical condition reported closely. Multiple LLTs converge into only one “preferred term” (PT) representing the next structural level. Several PTs can then be grouped using anatomical, pathological, physiological, etiological, or functional criteria in “High-Level Terms” (HLTs). HLTs can then be categorized in “High-Level Group Terms” (HLGTs). Finally, the highest-level terms of this classification are represented by the so-called “System Organ Classes” (SOCs), which provide a broader data overview. As far as seriousness was concerned, a case was defined as ‘serious’ when highlighted at least an ADR resulting in death, hospitalization, persistent or significant disability/incapacity, congenital anomaly/birth defect or conditions deemed as medically important by the reporter, prolonging hospitalization or being life-threatening. For the ADRs outcomes standardized terminology was used with ADRs classified as: ‘recovered/resolved’, ‘recovering/resolving’, ‘recovered/resolved with sequelae’, ‘not recovered/not resolved’, ‘fatal’, and ‘unknown’ on the bases of what was reported in the ICSR. The ADR expectedness was verified based on the Summary of Product Characteristics (SmPCs) available in the EMA database (31). If two or more ADR Symptoms reported in the same ICSR presented different outcomes a global outcome for the case described in the ICSR was computed using the “Lower Level of Resolution” methodology previously described by other authors (32).

### 2.4. Statistical analysis

For ICSRs characteristics comparisons, we used the Chi-square test and the U Mann–Whitney test for categorical and continuous variables, respectively. The distribution of variables was tested using Shapiro-Wilks and Kolmogorov–Smirnov tests. Continuous variables were reported as median values with associated interquartile ranges (IQRs). Categorical variables were synthesized as frequencies and percentages. ICSRs simultaneously involving a PP-based formulation and another SGA-LAI as suspected drugs were excluded from comparisons between the two groups. The Chi-square test was applied to evaluate differences for ADR characteristics between PP-related ICSRs and the reference group. Values of *p* < 0.001 were considered statistically significant.

Disproportionalities in the observed ADR frequencies for PP-related ICSRs compared to those of ICSRs presenting as suspected drugs other SGA-LAIs were evaluated by calculating the Reporting Odds Ratios (RORs) and associated 95% confidence intervals (95% CI). The statistical significance threshold was defined as 95% CI lower bound >1 in the presence of ≥3 reports per PP formulation. For ADR regrouping purposes, we used the standardized MedDRA^®^ queries (SMQs), which are groups of MedDRA^®^ terms related to a defined medical condition or area of interest (33). Regrouping terms by SMQs can be done by using either ‘narrow’ or ‘broad’ search strategies. For this analysis, we used the more narrow-scope approach, with terms characterized by a higher likelihood of representing the condition of interest (34). In addition, a sub-analysis using the Chi-square test methodology was performed to compare the ADR reporting frequencies between PP3M and PP1M. All the analyses were carried out using the Statistical Package for Social Science (SPSS, International Business Machines Corporation) Version 28.

## 3. Results

**Figure 1:**
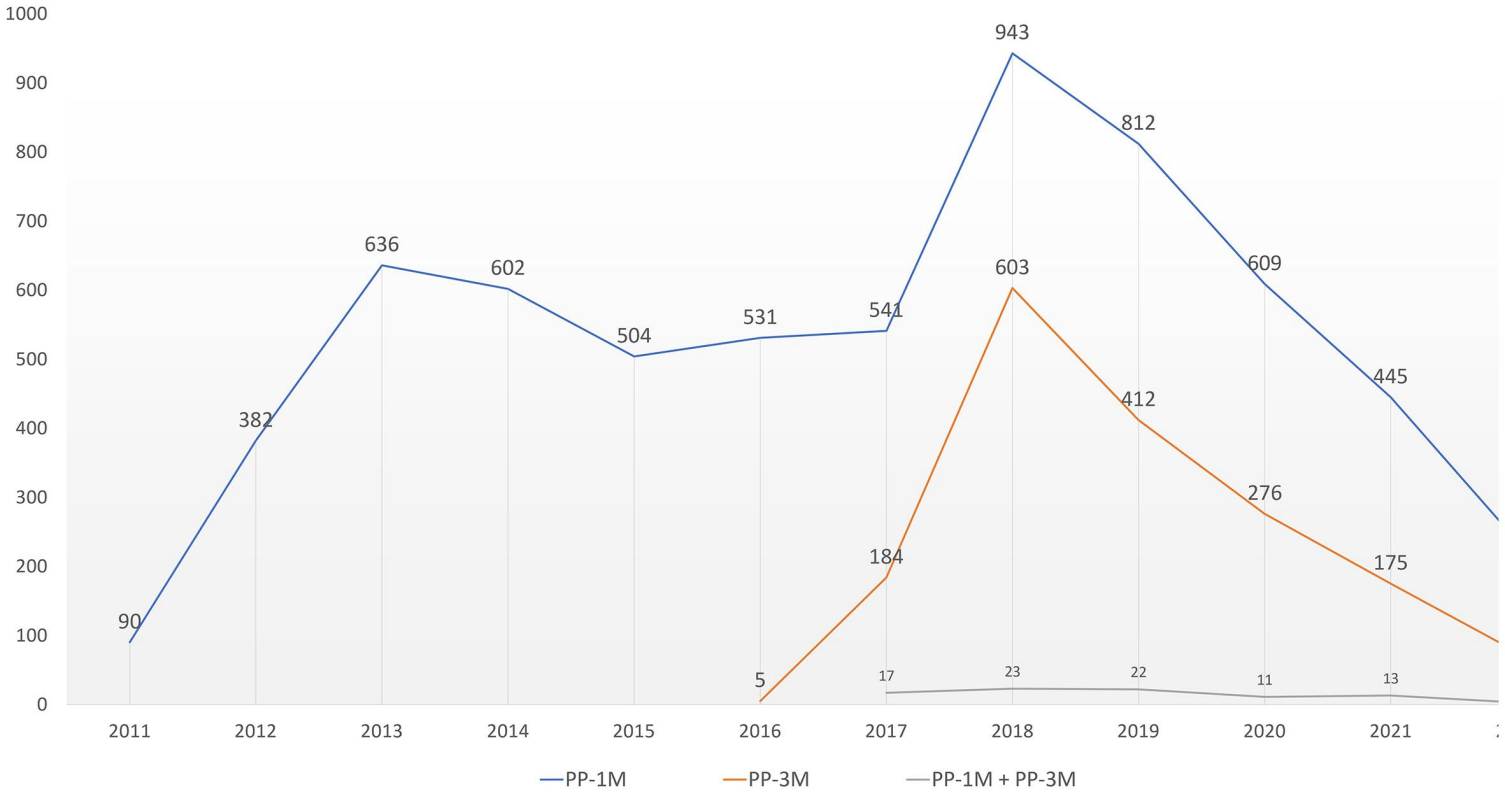
PP-related ICSRs temporal distribution. ICSRs, Individual Case Safety Reports; PP, paliperidone palmitate; PP1M, paliperidone palmitate 1-month; PP3M, paliperidone palmitate 3-month.

Overall, 20,226 ICSRs related to SGA-LAIs were retrieved from the EUDRAVigilance dataset during the observation period. Of those, 8,152 ICSRs indicated PP-based formulations as suspected culprit drugs. Among the PP-related reports, 6,332 (77.7%) presented as suspected drug PP1M and 1,731 (21.2%) PP3M, while 89 ICSRs indicated involvement of both PP formulations. As far as the ICSRs in the reference group were concerned, risperidone LAI was the SGA-LAI most frequently reported (*n* = 5,317; 43.7%), followed by aripiprazole LAI (*n* = 4,038; 33.2%) and olanzapine pamoate (*n* = 2,802; 23%). In the ICSRs for the reference group, 13 cases presenting referred to patients treated with multiple SGA-LAIs. In addition, 96 retrieved cases simultaneously involved a PP-based formulation and another SGA-LAI as suspected drugs. For PP-related ICSRs an initially steady trend was followed by a peak in 2018 (*n* = 1,569) after the introduction of PP3M in the market and a decreasing trend afterwards (Figure 1).

PP injection dose data were available in 6,312 (75.2%) ICSRs. The mean observed dose for PP-based formulations was 121,2 mg (±39 SD) for PP1M and 383,9 mg (±132.8 SD) for PP3M. Data for PP treatment duration were available in 430 (5.3%) ICSRs with a median PP treatment duration of 120 days for PP1M (IQR 31–337) and 244 (IQR 91–452) days for PP3.

Treatment indication information for PP based formulations were available in 57.1% (*n* = 4,655) of ICSRs. Among those, SCZ was the most frequently observed (*n* = 3,486; 74.9%), followed by psychotic disorders (*n* = 446; 9.6%), and schizoaffective disorders (*n* = 253; 5.4%). Table 1 summarizes the main characteristics of PP-related ICSRs compared to those related to the other SGA-LAIs.

**Table 1:** Characteristics of PP-related ICSRs compared to those of other SGAs related ICSRs.

Characteristic	PP-related ICSRs *n* = 8,152 (%)	Other SGA-LAIs (reference group; RG) ICSRs (RG) *n* = 12,170 (%)	*p*-value^a^^,^^b^ PP-related ICSRs versus RG
**Age categories (years)**			
Less than 18	60 (0.7)	146 (1.2)	0.007
18–64	5,255 (64.5)	8,306 (68.2)	0.083
65–85	460 (5.6)	751 (6.2)	0.524
More then 85	18 (0.2)	30 (0.2)	0.773
Not specified	2,359 (28.9)	2,937 (24.1)	–
**Sex**			
Male	4,814 (59.1)	6,764 (55.6)	0.001
Female	3,213 (39.4)	4,932 (40.5)	
Not specified	125 (1.5)	474 (3.9)	–
**Seriousness**			
Non-serious	2,888 (35.4)	2,079 (17.1)	<0.001
Serious	5,264 (64.6)	10,091 (82.9)	
Outcome			
Recovered/resolved	1,543 (18.9)	3,390 (27.9)	<0.001
Not recovered/not resolved	1,344 (16.5)	2,532 (20.8)	<0.001
Recovering/resolving	856 (10.5)	886 (7.3)	<0.001
Fatal	468 (5.7)	679 (5.6)	0.693
Recovered/resolved with sequelae	52 (0.6)	92 (0.8)	0.307
Not available	3,889 (47.7)	4,591 (37.7)	–

ICSRs, Individual Case Safety Reports; LAI, long-acting injectable; PP, paliperidone palmitate; RG, reference group; SGA, second-generation antipsychotic.

a *p*-values were calculated using the Chi-square test.

b ICSRs presenting both PP formulations and Other SGA LAIs as suspected drugs (*n* = 96) were excluded from the calculations.

Considering the suspected drugs other than PP, 36.8% (*n* = 3,082) of all PP-related ICSRs presented at least an additional suspected drug. A median value of 1 (IQR 1–2) for the number of co-reported suspected drugs was reported. Stratifying ICSRs by PP formulation, the number of co-reported suspected drugs remained constant for PP1M and PP3M-related ICSRs with the PP3M-related ones exhibited a narrower IQR (1-1). In qualitative terms the most frequently co-reported suspected drugs 65.4% (*n* = 1,311) belonged to the N05A ATC class (i.e., antipsychotics) namely, risperidone (*n* = 516; 39.4%), olanzapine (*n* = 125; 9.5%), and aripiprazole (*n* = 117; 8.9). Following the N05A was the N03A class (i.e., antiepileptics) (*n* = 120; 6%), with valproic acid (*n* = 71; 59.2%), clonazepam (*n* = 14; 11.7%), and lamotrigine (*n* = 10; 8.3%). After that, the N06A class drugs (i.e., antidepressants) had the higher frequency (*n* = 106; 5.3%), namely, escitalopram (*n* = 15; 14.2%), sertraline (*n* = 13; 12.3%), and paroxetine (*n* = 12; 11.3%). More details on the distribution of suspect drugs groups according to the ATC classification, per single PP-derived formulation is available in the Electronic Supplementary Material (ESM) Table 1.

In terms of ADR seriousness, 64.6% (*n* = 5,264) of PP-related ICSRs indicated at least one ADR classifiable as serious, less frequently than in the reference group (*n* = 10,091; 64.6%, *p* < 0.001). Outcome data were available in 52.3% of PP-related ICSRs. In detail 1,543 cases (36.2%) described one ADR deemed as “Recovered/Resolved,” 1,344 (31.5%) cases one labelled as “Not Recovered/Not Resolved,” and 856 (20.1%) one ADR that was still “Recovering/Resolving” at the time of the last available follow-up (Table 1).

ADRs observed in PP-related ICSRs mainly concerned the SOCs “Psychiatric disorders” (*n* = 2,898; 19.3%), “General disorders and administration site conditions” (*n* = 2,608; 17.4%), “Nervous system disorders” (*n* = 1946; 13.0%), “Injury, poisoning and procedural complications” (*n* = 1,321; 8.8%), and “Investigations” (*n* = 1,554 179; 7.9%).

ADRs labelled “Endocrine disorders” were more frequently reported in PP-related ICSRs, compared to the reference group (Table 2). The specific ADRs related to this SOC, classified at the MedDRA PT level for PP were hyperprolactinaemia (*n* = 226; 88.6%), followed by inappropriate antidiuretic hormone secretion (*n* = 9; 3.5%), hypothyroidism (*n* = 4; 1.6%), thyroid disorder (*n* = 3; 1.2%), and diabetes insipidus (*n* = 2; 0.8%).

**Table 2:** Relative ADRs frequencies observed in PP-related ICSRs formulations as compared to reference group ICSRs, stratified by system organ class.

System organ classes	PP-related^a^ ICSRs *N* = 8,056 (%^b^)	Other SGA-LAIs (reference group; RG) *N* = 12,170 (%^b^)	*p* value PP versus RG^a^
Blood and lymphatic system disorders	135 (1.7)	210 (1.7)	0.789
Cardiac disorders	353 (4.4)	742 (6.1)	<0.001
Congenital, familial and genetic disorders	17 (0.2)	22 (0.2)	0.631
Ear and labyrinth disorders	59 (0.7)	160 (1.3)	<0.001
Endocrine disorders	246 (3.1)	257 (2.1)	<0.001
Eye disorders	260 (3.2)	460 (3.8)	0.038
Gastrointestinal disorders	439 (5.4)	890 (7.3)	<0.001
General disorders and administration site conditions	2,576 (32)	4,100 (33.7)	0.011
Hepatobiliary disorders	70 (0.9)	116 (1)	0.539
Immune system disorders	65 (0.8)	98 (0.8)	0.990
Infections and infestations	295 (3.7)	470 (3.9)	0.465
Injury, poisoning and procedural complications	1,301 (16.1)	2,818 (23.2)	<0.001
Investigations	1,162 (14.4)	2,426 (19.9)	<0.001
Metabolism and nutrition disorders	335 (4.2)	813 (6.7)	<0.001
Musculoskeletal and connective tissue disorders	490 (6.1)	859 (7.1)	0.006
Neoplasms benign, malignant and unspecified (including cysts and polyps)	85 (1.1)	146 (1.2)	0.344
Nervous system disorders	1,904 (23.6)	4,602 (37.8)	<0.001
Pregnancy, puerperium and perinatal conditions	28 (0.3)	79 (0.6)	0.004
Product issues	76 (0.9)	264 (2.2)	<0.001
Psychiatric disorders	2,862 (35.5)	4,644 (38.2)	<0.001
Renal and urinary disorders	159 (2)	337 (2.8)	<0.001
Reproductive system and breast disorders	620 (7.7)	836 (6.9)	0.026
Respiratory, thoracic and mediastinal disorders	358 (4.4)	555 (4.6)	0.696
Skin and subcutaneous tissue disorders	339 (4.2)	545 (4.5)	0.358
Social circumstances	106 (1.3)	348 (2.9)	<0.001
Surgical and medical procedures	149 (1.8)	526 (4.3)	<0.001
Vascular disorders	244 (3)	732 (6)	<0.001

ADR, adverse drug reaction; ICSRs, Individual Case Safety Reports; PP, paliperidone palmitate; RG, reference group; SOC, system organ class.

a ICSRs presenting both categories of drugs as suspected (*n* = 96) were excluded from the calculations.

b For each SOC, the number of reports with at least one ADR related to the SOC are reported. The sum of the distribution of reports of ADRs by SOC (%) is higher than the total number of reports, since a single report could contain ADRs related to more than one SOC.

There were 468 ICSRs reporting fatal outcomes. Most of them (*n* = 303; 64.7%) regarded male patients, and 330 cases (70.5%) were in the 18 to 64 years age group. The number of reported suspected drugs other than PP in this ICSRs was higher when compared to all other PP related ICSR (2.4 ± 2.1 SD vs. 1.6 ± 1.3 SD; *p* < 0.001) without however, major differences in terms of the type of co-reported suspected drugs. In these ICSRs the most frequently observed ADRs were related to the MedDRA HLTs “Death and sudden death” (*n* = 181; 17.4%), “Suicidal and self-injurious behavior” (*n* = 103; 9.9%), “Ischemic coronary artery disorders (*n* = 26; 2.5%),” “Ventricular arrhythmias and cardiac arrest” and “Product administration errors and issues” (both with *n* = 24; 2.3%). Among the specific ADRs leading to fatal outcomes those observed with higher frequencies were, aside from death (*n* = 129; 21.8%) and sudden death (*n* = 47; 7.9%), completed suicide (*n* = 98; 16.5%), pulmonary embolism (*n* = 16; 2.7%), myocardial infarction (n = 14; 2.4%), cardiac failure (*n* = 13; 2.2%), and cardio-respiratory arrest (*n* = 12; 2%).

**Figure 2:**
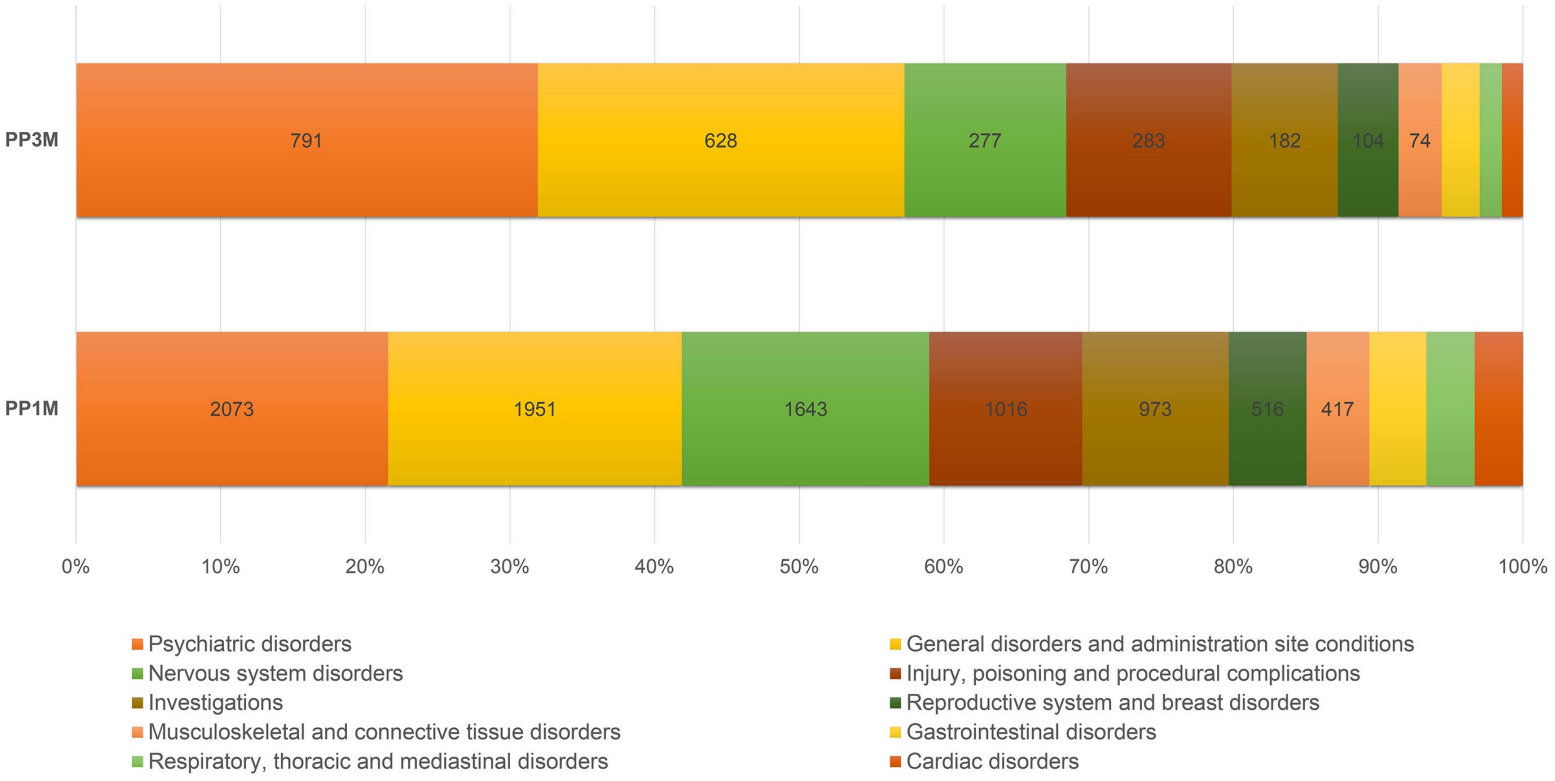
Relative reporting frequencies of ADRs belonging to the 10 most frequently observed SOCs. ADR, adverse drug reaction; PP1M, paliperidone palmitate 1-month; PP3M, paliperidone palmitate 3-month; SOCs, system organ classes.

Comparing to PP1M-related ICSRs, the PP3M-related ICSRs more frequently contained the SOCs “psychiatric disorders,” “general disorder and administration site conditions,” and “product issues” (*p* < 0.001). The relative reporting frequencies for the 10 major SOCs are reported in Figure 2, while full details are available in Table 3. In PP3M-related ICSRs, the specific ADRs more frequently reported as “psychiatric disorders” were SCZ (*n* = 174; 16.7%), psychotic disorder (*n* = 97; 9.3%), psychotic symptom (*n* = 69 6.6%), delusion (*n* = 54; 5.2%), psychiatric decompensation (*n* = 53; 5.1%), and anxiety (*n* = 47; 4.5%). The specific ADRs in PP3M ICSRs, relative to “general disorder and administration site conditions” were mainly “drug ineffective” (*n* = 158; 20.8%), “condition aggravated” (*n* = 101; 13.3%), “malaise” (*n* = 49; 6.5%), “fatigue” (*n* = 48; 6.3%), and “injection site pain” (*n* = 36; 4.7%). While for the SOC “product issues” the ADRs observed with the highest frequency in PP3M ICSRs were “device occlusion” (*n* = 7; 17.9%), “syringe issue” (*n* = 6; 15.4%), “product complaint” (*n* = 6; 15.4%), “needle issue” (*n* = 5; 12.8%), and “product quality issue” (*n* = 4; 10.3%). More details on specific ADRs related to each SOC at the MedDRA PT level is available in Electronic Supplementary Material (ESM) Table 2.

**Table 3:** Relative ADRs frequencies observed in PP3M-related ICSRs formulations as compared to PP1M-related ICSRs, stratified by system organ class.

System organ classes	PP3M-related^a^ ICSRS	PP1M-related^a^ ICSRs (RG)	*p* value PP3M versus PP1M^a^
	*N* = 1,731 (%^b^)	*N* = 6,332 (%^b^)	
Blood and lymphatic system disorders	19 (1.1)	115 (1.8)	0.038
Cardiac disorders	36 (2.1)	320 (5.1)	<0.001
Congenital, familial and genetic disorders	4 (0.2)	13 (0.2)	0.836
Ear and labyrinth disorders	13 (0.8)	46 (0.7)	0.915
Endocrine disorders	34 (2)	213 (3.4)	0.003
Eye disorders	37 (2.1)	230 (3.6)	0.002
Gastrointestinal disorders	65 (3.8)	379 (6)	<0.001
General disorders and administration site conditions	628 (36.3)	1951 (30.8)	<0.001
Hepatobiliary disorders	7 (0.4)	63 (1)	0.019
Immune system disorders	3 (0.2)	63 (1)	0.001
Infections and infestations	37 (2.1)	259 (4.1)	<0.001
Injury, poisoning and procedural complications	283 (16.3)	1,016 (16)	0.761
Investigations	182 (10.5)	973 (15.4)	<0.001
Metabolism and nutrition disorders	47 (2.7)	295 (4.7)	<0.001
Musculoskeletal and connective tissue disorders	74 (4.3)	417 (6.6)	<0.001
Neoplasms benign, malignant and unspecified (including cysts and polyps)	14 (0.8)	72 (1.1)	0.239
Nervous system disorders	277 (16)	1,643 (25.9)	<0.001
Pregnancy, puerperium and perinatal conditions	4 (0.2)	24 (0.4)	0.354
Product issues	36 (2.1)	40 (0.6)	<0.001
Psychiatric disorders	791 (45.7)	2073 (32.7)	<0.001
Renal and urinary disorders	32 (1.8)	127 (2)	0.677
Reproductive system and breast disorders	104 (6)	516 (8.1)	0.003
Respiratory, thoracic and mediastinal disorders	38 (2.2)	321 (5.1)	<0.001
Skin and subcutaneous tissue disorders	45 (2.6)	296 (4.7)	<0.001
Social circumstances	19 (1.1)	85 (1.3)	0.424
Surgical and medical procedures	22 (1.3)	126 (2)	0.048
Vascular disorders	32 (1.8)	216 (3.4)	0.001

ADR, adverse drug reaction; ICSRs, Individual Case Safety Reports; PP, paliperidone palmitate; PP1M, paliperidone palmitate 1-month; PP3M, paliperidone palmitate 3-month; SOC, system organ class.

a ICSRs presenting both categories drugs as suspected (*n* = 89) were excluded from the calculations.

b For each SOC, the number of reports with at least one ADR related to the SOC are reported. The sum of the distribution of reports of ADRs by SOC (%) is higher than the total number of reports, since a single report could contain ADRs related to more than one SOC.

Significantly disproportionate reporting, for PP-related reports compared to the reference group, was observed for SMQs “Sexual Dysfunctions” (ROR = 1.45; 95% CI 1.23–1.70), “Haemodynamic oedema, effusions and fluid overload” (ROR = 1.42; 1.18–1.70), as well as “Fertility disorders” (ROR = 2.69; 1.51–4.80) (Table 4). In terms of secondary SMQs only “Parkinson-like events” (ROR = 1.27; 1.06–1.53) were disproportionately reported for PP formulations compared to the reference group Electronic Supplementary Material (ESM) (Table 3).

**Table 4:** Reporting odds ratios for PP-related ICSRs as compared to RG using standardized MedDRA queries.

Individual SMQ^a^	PP related ICSRs *N* = 8,056^b^	95% CI	ROR
Psychosis and psychotic disorders	1,331	0.85–0.99	0.92
Medication errors	642	0.92–1.14	1.03
Lack of efficacy/effect	640	0.97–1.19	1.07
Extrapyramidal syndrome	601	0.65–0.79	0.72
Depression and suicide/self-injury	430	0.68–0.86	0.76
Sexual dysfunction	287	1.23–1.7	1.45
Hypersensitivity	264	0.81–1.11	0.95
Embolic and thrombotic events	253	0.68–0.92	0.79
Gastrointestinal nonspecific inflammation and dysfunctional conditions	231	0.57–0.78	0.66
Haemodynamic oedema, effusions and fluid overload	229	1.18–1.7	1.42
Oropharyngeal disorders	178	0.68–0.99	0.82
Hepatic disorders	158	0.72–1.07	0.88
Hostility/aggression	143	0.41–0.61	0.50
Haematopoietic cytopenias	130	0.81–1.26	1.01
Accidents and injuries	127	0.54–0.83	0.67
Cardiac arrhythmias	125	0.69–1.07	0.86
Shock	121	0.81–1.3	1.03
Hyperglycaemia/new onset diabetes mellitus	115	0.34–0.51	0.42
Noninfectious encephalopathy/delirium	113	0.54–0.84	0.67
Neuroleptic malignant syndrome	110	0.71–1.15	0.91
Ocular motility disorders	98	0.73–1.21	0.94
Haemorrhages	91	0.6–1	0.77
Convulsions	81	0.41–0.69	0.53
Generalised convulsive seizures following immunisation	79	0.42–0.71	0.55
Hypertension	74	0.21–0.35	0.27
Pregnancy and neonatal topics	71	0.44–0.75	0.57
Angioedema	70	0.55–0.97	0.73
Central nervous system vascular disorders	70	0.53–0.93	0.70
Malignancies	69	0.65–1.19	0.88
Drug abuse, dependence and withdrawal	67	0.64–1.17	0.86
Rhabdomyolysis/myopathy	58	0.96–1.96	1.37
Torsade de pointes/QT prolongation	58	0.93–1.89	1.33
Hearing and vestibular disorders	55	0.4–0.75	0.55
Ischaemic heart disease	55	0.47–0.9	0.65
Acute renal failure	48	0.68–1.41	0.98
Dyslipidaemia	48	0.42–0.82	0.59
Immune-mediated/autoimmune disorders	44	0.79–1.73	1.17
Infective pneumonia	44	0.51–1.05	0.73
Hyponatraemia/SIADH	40	0.64–1.43	0.96
Cardiac failure	38	0.61–1.36	0.91
Noninfectious diarrhoea	37	0.52–1.15	0.78
Gastrointestinal perforation, ulceration, haemorrhage or obstruction	36	0.5–1.11	0.74
Fertility disorders	32	1.51–4.8	2.69
Respiratory failure	29	0.4–0.95	0.62
Periorbital and eyelid disorders	26	0.69–1.93	1.16
Acute central respiratory depression	25	0.38–0.97	0.61
Anaphylactic reaction	22	0.53–1.52	0.90
Peripheral neuropathy	22	0.66–2	1.15
Conjunctival disorders	20	0.85–2.98	1.59
Biliary disorders	19	0.36–1.04	0.61
Acute pancreatitis	18	0.42–1.29	0.73
Agranulocytosis	18	0.54–1.76	0.97
Dehydration	18	0.52–1.69	0.94
Lacrimal disorders	18	0.61–2.09	1.13
Taste and smell disorders	16	0.53–1.9	1.01
Chronic kidney disease	15	0.28–0.9	0.50

COVID-19, coronavirus disease; ICSRs, Individual Case Safety Reports; PP, paliperidone palmitate; SIADH, syndrome of inappropriate antidiuretic hormone secretion.

a Primary SMQs with less than 15 associated cases have been excluded from this table. Full details regarding primary and secondary SMQs are available in ESM Table 3.

b ICSRs distribution by SMQ is not mutually exclusive.

## 4. Discussion

### 4.1. General ICSRs characteristics and frequently observed ADRs

Among SGA-LAIs PP is the only one currently available not only in a monthly but also a quarterly and more recently a half-yearly administration formulation, thus making it one of the most interesting therapeutic options to maintain treatment adherence in long-term treatment of patients suffering from SCZ (35). Therefore, the constant rising number of ICSRS per year observed in our analysis reflects this continuous level of increasing attention on PP since its market introduction. Furthermore, the ICSRs reporting peak of 2018 following the market introduction of the PP3M formulation shows that this event also had an increasing effect on the yearly reporting frequency of the PP1M-related ICSRs. Thus, we expect an increase for ADRs reports in the coming years following the introduction of the six-monthly formulation as use and clinical experience increase. It must be pointed out however, that the market approval process of SGA-LAIs has not happened simultaneously in all the countries covered by the EUDRAVigilance database. Moreover, differences in the availability of these drugs still persist today.

As far as patient characteristics are concerned, the observed differences in terms of reported patients’ age, between PP and RG-related ICSRs, seem to be in line with routinely clinical practice. PP-based formulations have been introduced more recently than the other LAIs which makes them less likely to be selected by clinicians for treating patients before the age of 18. Also, the lack of EMA-approved indications for their use in pediatric patients limits the use of both PP1M and PP3M in this context (36, 37). The observed differences in terms of reported patient sex may be more attributable to ADRs commonly associated with PP than to effective sex differences in tolerability. In fact, ADRs related to prolactin increases are frequently observed with PP, but they could be considered more in women as they are clinically more impactful (e.g., amenorrhea). This could lead to considering more carefully the administration of PP based in women and by consequence to an observation bias. However, our findings prevent us from formulating any conclusion in this regard.

The data regarding the types of co-reported suspected drugs highlight that almost 40% of ICSRs involved at least one co-medication. Most of the observed co-reported suspected drugs were oral antipsychotics. Adding an oral antipsychotic to LAI-based therapeutic regimens is a common practice in the initial phases to mitigate risks related to the slow release of the LAI formulations (38). Also, antipsychotic polypharmacy (APP) is frequently used in clinical practice. It has been estimated that 10–20% of outpatients and up to 40% of inpatients diagnosed with SCZ are treated with APP mainly as augmentation (39). The frequent combination of PP with mood stabilizers and/or antidepressants and benzodiazepines in the relevant ICSRs highlights the risks that may emerge in the context of such therapeutic regimens (40).

Regarding seriousness of ADRs, PP-related ICSRs were less frequently serious compared to other SGA-LAIs. This is in line with findings from other types of literature that highlighted overall good tolerability for PP when compared to other SGA-LAIs (41). As far as ADR outcomes are concerned, significantly higher (*p* < 0.001) frequencies of cases describing ADRs deemed as “recovering/resolving” were observed in PP-related ICSRs when compared to the reference group. Significant differences but in a diminutive sense were observed for ADR cases with a complete recovery and with reactions not resolved at the time of the last follow-up between ICSRs PP-related and in the reference group. This data correlates well with the type of observed ADRs in PP-related ICSRs as several of the most frequently observed ADRs such as those relative to “Psychiatric disorders” and “Nervous system disorders” are generally characterized by long resolution periods (e.g., literature sources report a median of 91 days for extrapyramidal symptoms) (42, 43).

In terms of specific ADRs, “Psychiatric disorders” related ones were mainly associated to the onset of psychotic episodes, anxious manifestations, and insomnia (ESM Table 2). While anxiety and insomnia are listed as ADRs frequently associated with PP (36, 37), some considerations must be made regarding symptoms related to SCZ reported as suspected ADRs. Among the ICSRs reporting “schizophrenia” as one of the described specific ADRs, 22.2% presented at least an ADR classifiable within the high-level term “therapeutic and non-therapeutic responses (e.g., Drug ineffective, Treatment noncompliance, Therapeutic product effect decreased) and 12.3% at least one ADR relative to “Product administration errors and issues” (e.g., Inappropriate schedule of product administration; Product dose omission issue; Incorrect dose administered). Furthermore, literature sources indicate that 20 to 30% of patients affected by SCZ are known to not respond to treatment with antipsychotics (44, 45), and data suggest a form of secondary treatment-resistant SCZ (46, 47). Considering this, we could reasonably say that most of these ADRs are more likely to derive from insufficient therapeutic control or relapses of pre-existing diseases rather than from exposure to the drug. This is also confirmed to what is reported for the General disorders and administration site conditions SOC, in which ADRs such as “drug ineffective” and “Condition aggravated” were characterized by the higher frequencies of reporting.

In ICSRs reporting fatal cases, the most frequently reported specific ADRs described suicidal and self-injurious behaviors. These behaviors have been associated with SCZ; a recent study has estimated an increase of 4.5 times of the incidence of these conditions over the general population for patients with SSDs (48). The risk factors for these types of manifestations are highly complex and range from demographic characteristics to psychosocial factors (49). This complexity requires an in-depth case-by-case assessment approach to properly evaluate these reactions, which could require a different study design to investigate. Other common types of ADRs observed in this subgroup of ICSRs included pulmonary embolism and cardiac failure. These ADRs have already been reported in the context of antipsychotic treatment data (50). Data regarding a link between paliperidone and pulmonary thromboembolism, however, are limited to few cases (51, 52). Moreover, the underlying mechanism of this ADR is still largely unknown, although some hypotheses regarding prolactin and its potential role as a platelet aggregation coactivator have been proposed (53). However, the influence of other factors such as obesity, increased levels of antiphospholipid antibodies, and hyperhomocysteinemia remains unclear. On this matter, some authors suggested that using aripiprazole would be preferable in patients presenting possible risk factors (52), but the clinical experience in this sense remains limited. In addition, 8.9% of the total neuroleptic malignant syndrome (NMS) cases observed (*n* = 113) presented a fatal outcome for the patient. This severe idiosyncratic reaction is linked to the administration of dopamine-blocking agents such as antipsychotics. It presents with symptoms such as fever, muscle rigidity, alterations in mental status, and autonomic functioning (54–56). Since LAI antipsychotics cannot be cleared quickly from the patient’s system, using these formulations can be perceived by the clinicians as limiting in terms of NMS management options, negatively impacting their perceived safety (57). Some recent retrospective studies have, however, estimated a low incidence of NMS cases over the antipsychotic-treated population, equal to 1.99 (95%CI 1.98–2.00) per 10,000 person-years, without any statistically significant differences between oral and LAI antipsychotics formulations (58, 59). This is in line with the results from our analysis, showing no disproportionality in the reporting of NMS between ICSRs PP-related and in the reference group (Table 4).

### 4.2. Disproportional ADRs

#### 4.2.1. Sexual and fertility disorders

Our analysis has highlighted an increased probability of reporting for ADRs relative to the SMQs “sexual dysfunction” and “fertility disorder” between PP and the reference group. Sexual dysfunctions are commonly associated with antipsychotics. Literature sources state that up to 75% of treated patients experienced sexual dysfunction (60). However, their incidence could be underestimated due to the reluctancy of patients and physicians to spontaneously discuss and report these kind of reactions (61). These ADRs have multifactorial processes regarding underlying mechanisms. One of the most widely embraced factors is the increase in prolactin levels resulting from the antagonistic action on D2 dopamine receptors that characterizes antipsychotics. Dopaminergic receptors in the hypothalamic tuberoinfundibular tract act as inhibitors for prolactin secretion; thus, inhibition of dopamine D2 receptors in this tract increases prolactin release. This increase results in an inhibition of the release of follicle-stimulating and luteinizing hormones from the pituitary gland. With consequent low gonadal steroids and hypogonadism (62). The impact of these ADRs cannot be underestimated as they can negatively influence patient’s quality of life and potentially reduce treatment compliance (63, 64). The importance of these aspects is particularly central for LAI-treated patients, considering that candidate patients for LAI treatment are usually middle-aged adults, already stabilized in treatment with an AP, for which clinicians seek therapies that could help them improve their quality of life and regain as much social functionality as possible (65). Prolactin-related ADRs could also limit the use of these LAIs in populations of youth with serious mental illness who are at risk for relapse, for which SGA-LAIs could represent an effective treatment strategy (66). A previous prospective study highlighted significant increases in mean prolactin values in risperidone-treated young patients, with long-term consequences of these ADRs still on patients’ development to be clarified (66, 67).

#### 4.2.2. Oedema related ADRs

ADRs relative to various forms of peripheral oedema constituted the vast majority of the SMQ “Haemodynamic oedema, effusions and fluid overload” for which a higher probability of reporting in PP-related when compared to RG-related ICSRs emerged from our analysis. These ADRs are already acknowledged as class effects related to the administration of SGA-LAIs. The mechanism underlying this type of ADRs remains unclear; however, several hypotheses have been formulated. Paliperidone being chemically a derivate of risperidone acts with a similar mechanism by blocking the serotonin (5-hydroxytryptamine, 5-HT) 5HT_2_, Dopaminergic D2, Adrenergic α1, α2 and histaminergic H1 receptors (68). The blockage of α1 receptors results in vasodilation with a consequential increase in hydrostatic pressure in the capillaries that could facilitate the onset of oedema (69). Also, the antagonistic action on 5HT2 receptors could be associated to oedema due to the increase in cyclic adenosine monophosphate concentrations, leading to the relaxation of vascular smooth muscle (70).

#### 4.2.3. Extrapyramidal disorders

Regarding ADRs related to nervous system no disproportionality in primary reporting was reported for the PP-related ICSRs compared to the reference range. However, secondary reports of Extrapyramidal syndrome and Parkinson-like events were more frequent for PP compared to the reference group. However, in these ICSRs there was a higher number of co-reported suspected drugs for extrapyramidal syndrome and Parkinson-like events compared to the rest of PP-related ICSRs (2.2 ± 1.8 SD vs. 1.6 ± 1.3 SD; *p* = 0.014). When repeating the ROR calculations including on ICSRs with only one suspected drug (either PP or other SGA-LAI) we did not detect disproportionality between PP and the reference group [PP cases = 98; ROR = 1.19 (95%CI: 0.91–1.57)]. Furthermore, the most frequently reported drugs other than PP in these reports were other antipsychotics. Considering these data, we can reasonably assume pharmacodynamic interactions in combination therapies underlying the risk of these ADRs.

#### 4.2.4. Other considerations

The observed disproportionalities in ADR reporting probability while being mostly in line with what is already known about paliperidone-based formulations, might seem puzzling at first since the 44% of ICSRs in the reference group presented LAI formulations of risperidone, which is a chemical precursor of paliperidone (9-hydroxyrisperidone), as a suspected drug. However, risperidone differs substantially from paliperidone from a pharmacokinetic standpoint. In fact, the not negligible fist passage effect, the presence of other metabolites (7-hydroxyrisperidone), and possible influences of cytochrome P450-2D6 and 3A4 individual efficiency status, all represent differentiating factors between the two drugs (71). It has been pointed out by several literature sources that these differences could significantly impact the safety and tolerability profile of these two drugs, as well as provide a different efficacy profile in clinical practice (72, 73). In addition to that, the relative novelty of PP-based formulations compared to risperidone LAI could constitute an attention-increasing factor for ADRs already well-known in previously introduced LAIs for such as those regarding sexual disorders and extrapyramidal manifestations.

### 4.3. ADR reporting patterns’ differences between PP1M and PP3M

Some differences in terms of relative reporting frequencies were noticed between the two PP formulations. Increased reporting frequencies in relation to the SOCs “psychiatric disorders,” “general disorder and administration site conditions,” and “product Issues” were observed for PP3M-related ICSRs when compared to the PP1M-related ones. The reporting of product issues could be linked to the relative novelty of PP3M compared to PP1M and the resulting limited clinical experience with PP3M. A recently marketed drug could be, in fact, more prone to initial product-related issues than a long-time marketed one. In this sense, it must be considered that currently, no meaningful Therapeutic Drug Monitoring (TDM) data are available for PP3M (74). However, from a recently published prospective study, no significant differences were observed for PP3M compared to PP1M in terms of safety (75). In addition, it is well known that in the initial phases of market presence, the attention reserved to the safety and tolerability aspects of a drug is higher. Potentially, the tendency of clinicians to propose newer treatments to patients that have performed well with existing options also needs to be considered (75). This underlines the necessity of further prospective studies involving large patient cohorts and clinicians more directly to properly assess these differences.

## 5. Strengths and limitations

To the best of our knowledge, this is one of the first pharmacovigilance studies to evaluate the safety and tolerability profiles of PP-based formulation using data from a European scale pharmacovigilance database.

Considering the relatively recent approval of PP3M and given the general paucity of real-world derived safety data for PP-based LAI formulations, data deriving from large scale SRS databases analyses can contribute to a better characterization of their safety profiles.

Although our findings provide a comprehensive perspective in the evaluation of PP-related ADRs, the results of the present study should be interpreted in the light of some limitations.

First, the granularity of data provided by the EUDRAVigilance platform is limited and frequently managed in a categorical fashion. We acknowledge that we followed a conservative approach in case of lack of sufficient data, frequently leading to case exclusion or downgrade of reported items if information was not consistent. In addition to that, public data access does not allow to use all other drugs reported in the EUDRAVigilance database as a reference group as for other datasets (76). Moreover, we acknowledge that the publicly accessible EUDRAVigilance data level did not allow to access to detailed information about the reporting country, for privacy reasons. We were therefore unable to differentiate the results by reporting country. Likewise, FGA-LAI were not used as a reference group due to the lack of pharmacovigilance data related to the first years of their market presence. Additionally, the provided data limited considerations regarding aspects such as the presence of multiple suspected drugs in ICSRs. It also has to be pointed out that the retrieval of ICSRs regarding formulations with limited geographical availability was not possible due to database limitations.

Second, we performed a retrospective evaluation of cases reported by clinicians without the homogeneous structure of a single research protocol by applying a cluster analysis method not foreseen at the time of original reporting to the EUDRAVigilance platform. This makes secondary analysis of these data speculative, although the use of large pharmacovigilance databases inherently presents this limitation without necessarily limiting the validity of the conclusions.

Third, pharmacovigilance data should be read considering some technical concerns, including under-reporting compared to global clinical population and difficulty in identifying confounders. Indeed, this implies that the ADRs reported may represent only a partial, probably under-representative, percentage of all ADRs which occur in everyday clinical practice. Also, the lack of data related to the number of patients effectively treated with these drugs within the considered period (i.e., the denominator of the incidence fraction) does not allow incidence calculations.

Thus, future prospective clinical studies using a longitudinal design are required to improve the understanding of tolerability and security profile of PP1M, PP3M, and PP6M.

Similarly, further large-scale pharmacovigilance studies of international datasets, and with full access to Level 2A EUDRAVigilance data (77), are required to provide a more reliable estimate of incidence, clinical characteristics, and outcomes of PP-related ADRs compared to other LAIs.

## 6. Conclusion

In light of pharmacoepidemiological trends, there is an urgent need to understand SGA-LAI-related ADRs. Compared to other SGA-LAIs increased probabilities of reporting for ADR categorized as referring to the endocrine system impacting patient sexuality and fertility were observed for PP formulations. Also, some clinically irrelevant differences in the ICSRs reporting pattern between PP1M and PP3M emerged requiring further investigation as clinical experience with PP3M increases. The results presented in this work do not discourage the prescription of SGA-LAI formulations but aim to enhance their safety.

## Data availability statement

The original contributions presented in the study are included in the article/Supplementary Material, further inquiries can be directed to the corresponding author/s.

## Ethics statement

Ethical review and approval was not required for the study on human participants in accordance with the local legislation and institutional requirements. Written informed consent from the participants’ legal guardian/next of kin was not required to participate in this study in accordance with the national legislation and the institutional requirements.

## Author contributions

GC and MAB retrieved and analyzed the data. GC and RdF drafted the paper and agreed to be accountable for all aspects of the work, ensuring that questions related to the accuracy or integrity of any part of the work are investigated appropriately. ES, GS, and PMC revised the paper for important intellectual content. ES, GS, and PDF approved the final version of the manuscript to be published. ES, GS, PMC, GC, and RdF developed the concept. All authors contributed to the article and approved the submitted version.

## Conflict of interest

The authors declare that the research was conducted in the absence of any commercial or financial relationships that could be construed as a potential conflict of interest.

## Publisher’s note

All claims expressed in this article are solely those of the authors and do not necessarily represent those of their affiliated organizations, or those of the publisher, the editors and the reviewers. Any product that may be evaluated in this article, or claim that may be made by its manufacturer, is not guaranteed or endorsed by the publisher.
