# Supplementary material for: Tolerability profile of paliperidone palmitate formulations: A pharmacovigilance analysis of the EUDRAVigilance database
Source: Front Psychiatry. 2023 Apr 6;14:1130636. doi: 10.3389/fpsyt.2023.1130636 (PMC10116827; doi:10.3389/fpsyt.2023.1130636)
Supplement: Supplementary file 1 [file Table_1.docx]

Supplementary Material

Tolerability Profile of Paliperidone Palmitate Formulations:

A Pharmacovigilance Analysis of The EUDRAVigilance Database

**Giuseppe Cicala^*^, Renato de Filippis, Maria Antonietta Barbieri, Paola Maria Cutroneo, Pasquale De Fazio, Georgios Schoretsanitis, Edoardo Spina**

*** Correspondence:** Giuseppe Cicala: gcicala@unime.it

**ESM Table 1.** Suspected drugs other than PP in PP-related ICSRs.

| **ATC Class and ATC level III code** | **Drugs in PP1M-related ICSRs**  **n (%)** | **Drugs in PP3M-related ICSRs**  **n (%)** | **Drugs in ICSRs related to both PP1M and PP3M**  **n (%)** | **Drugs in all PP-related ICSRs**  **n (%)** |
| --- | --- | --- | --- | --- |
| **N - Nervous System** | **1,681 (91.6)** | **132 (91)** | **22 (95.7)** | **1,835 (91.6)** |
| N05A | 1,208 (71.9) | 86 (65.2) | 17 (77.3) | 1,311 (71.4) |
| N03A | 112 (6.7) | 5 (3.8) | 3 (13.6) | 120 (6.5) |
| N06A | 91 (5.4) | 15 (11.4) | - | 106 (5.8) |
| N05B | 96 (5.7) | 8 (6.1) | - | 104 (5.7) |
| N05C | 70 (4.2) | 4 (3) | - | 74 (4) |
| N04A | 63 (3.7) | 7 (5.3) | 2 (9.1) | 72 (3.9) |
| N02B | 11 (0.7) | 3 (2.3) | - | 14 (0.8) |
| N02A | 10 (0.6) | 1 (0.8) | - | 11 (0.6) |
| N07B | 8 (0.5) | 1 (0.8) | - | 9 (0.5) |
| N06B | 5 (0.3) | 2 (1.5) | - | 7 (0.4) |
| N01B | 4 (0.2) | - | - | 4 (0.2) |
| N04B | 1 (0.1) | - | - | 1 (0.1) |
| N07X | 1 (0.1) | - | - | 1 (0.1) |
| N07A | 1 (0.1) | - | - | 1 (0.1) |
| **C – Cardiovascular System** | **46 (2.5)** | **3 (2.1)** | **-** | **49 (2.4)** |
| C07A | 12 (26.1) | 1 (33.3) | - | 13 (26.5) |
| C10A | 11 (23.9) | 1 (33.3) | - | 12 (24.5) |
| C09C | 6 (13) | - | - | 6 (12.2) |
| C09A | 4 (8.7) | 1 (33.3) | - | 5 (10.2) |
| C01B | 3 (6.5) | - | - | 3 (6.1) |
| C09B | 2 (4.3) | - | - | 2 (4.1) |
| C03D | 2 (4.3) | - | - | 2 (4.1) |
| C08C | 2 (4.3) | - | - | 2 (4.1) |
| C01C | 2 (4.3) | - | - | 2 (4.1) |
| C02A | 1 (2.2) | - | - | 1 (2) |
| C01D | 1 (2.2) | - | - | 1 (2) |
| **A – Alimentary Tract and Metabolism** | **35 (1.9)** | **3 (2.1)** | **-** | **38 (1.9)** |
| A10B | 10 (28.6) | 1 (33.3) | - | 11 (28.9) |
| A06A | 5 (14.3) | - | - | 5 (13.2) |
| A02A | 4 (11.4) | - | - | 4 (10.5) |
| A02B | 4 (11.4) | - | - | 4 (10.5) |
| A03F | 2 (5.7) | 1 (33.3) | - | 3 (7.9) |
| A11H | 2 (5.7) | - | - | 2 (5.3) |
| A07X | 1 (2.9) | - | - | 1 (2.6) |
| A05A | 1 (2.9) | - | - | 1 (2.6) |
| A10A | - | 1 (33.3) | - | 1 (2.6) |
| A11D | 1 (2.9) | - | - | 1 (2.6) |
| A12B | 1 (2.9) | - | - | 1 (2.6) |
| A04A | 1 (2.9) | - | - | 1 (2.6) |
| A07E | 1 (2.9) | - | - | 1 (2.6) |
| A01A | 1 (2.9) | - | - | 1 (2.6) |
| A07B | 1 (2.9) | - | - | 1 (2.6) |
| **R – Respiratory System** | **13 (0.7)** | **1 (0.7)** | **-** | **14 (0.7)** |
| R06A | 9 (69.2) | 1 (100) | - | 10 (71.4) |
| R01A | 2 (15.4) | - | - | 2 (14.3) |
| R03D | 1 (7.7) | - | - | 1 (7.1) |
| R03B | 1 (7.7) | - | - | 1 (7.1) |
| **V - Various** | **13 (0.7)** | **1 (0.7)** | **-** | **14 (0.7)** |
| V03A | 12 (92.3) | 1 (100) | - | 13 (92.9) |
| V10X | 1 (7.7) | - | - | 1 (7.1) |
| **B – Blood and Blood forming Organs** | **10 (0.5)** | **1 (0.7)** | **1 (4.3)** | **12 (0.6)** |
| B01A | 8 (80) | 1 (100) | 1 (100) | 10 (83.3) |
| B03B | 2 (20) | - | - | 2 (16.7) |
| **J - Antiinfectives For Systemic Use** | 9 (0.5) | 1 (0.7) | - | 10 (0.5) |
| J05A | 4 (44.4) | 1 (100) | - | 5 (50) |
| J01C | 3 (33.3) | - | - | 3 (30) |
| J01F | 1 (11.1) | - | - | 1 (10) |
| J01D | 1 (11.1) | - | - | 1 (10) |
| **L - Antineoplastic and Immunomodulating Agents** | **8 (0.4)** | **-** | **-** | **8 (0.4)** |
| L01X | 3 (37.5) | - | - | 3 (37.5) |
| L04A | 1 (12.5) | - | - | 1 (12.5) |
| L02B | 1 (12.5) | - | - | 1 (12.5) |
| L01C | 1 (12.5) | - | - | 1 (12.5) |
| L01B | 1 (12.5) | - | - | 1 (12.5) |
| L01D | 1 (12.5) | - | - | 1 (12.5) |
| **D - Dermatologicals** | 7 (0.4) | - | - | 7 (0.3) |
| D04A | 6 (85.7) | - | - | 6 (85.7) |
| R06A | 1 (14.3) | - | - | 1 (14.3) |
| **M - Musculo-Skeletal System** | **7 (0.4)** | **-** | **-** | **7 (0.3)** |
| M04A | 3 (42.9) | - | - | 3 (42.9) |
| M03B | 2 (28.6) | - | - | 2 (28.6) |
| M01A | 1 (14.3) | - | - | 1 (14.3) |
| M02A | 1 (14.3) | - | - | 1 (14.3) |
| **G - Genito Urinary System and Sex Hormones** | **4 (0.2)** | **1 (0.7)** | **-** | **5 (0.2)** |
| G03A | 2 (50) | - | - | 2 (40) |
| G04B | 1 (25) | - | - | 1 (20) |
| G01A | 1 (25) | - | - | 1 (20) |
| G02C | - | 1 (100) | - | 1 (20) |
| **H - Systemic Hormonal Preparations. Excl. Sex Hormones And Insulins** | **3 (0.2)** | **2 (1.4)** | **-** | **5 (0.2)** |
| H03A | 3 (100) | 1 (50) | - | 4 (80) |
| H05B | - | 1 (50) | - | 1 (20) |

ATC: Anatomical Therapeutic Chemical Classification System; ICSRs: Individual Case Safety Reports; PP: paliperidone palmitate; PP1M: paliperidone palmitate 1-month; PP3M: paliperidone palmitate 3-month.

**ESM Table 2.** ADRs at MedDRA Preferred Terms level regrouped by SOC for all PP-based Formulations.

| **ADRs by SOC*** | **N in**  **PP1M ICSRs** | **N in**  **PP3M ICSRs** | **N in**  **PP1M+PP3M ICSRs** | **N in**  **All PP-related ICSRs** |
| --- | --- | --- | --- | --- |
| **Blood and lymphatic system disorders** |  |  |  |  |
| Leukopenia | 30 | 8 | - | 38 |
| Neutropenia | 30 | 4 | - | 34 |
| Thrombocytopenia | 22 | 4 | - | 26 |
| Anaemia | 16 | - | 1 | 17 |
| Leucocytosis | 10 | 1 | - | 11 |
| Pancytopenia | 7 | 1 | - | 8 |
| **Cardiac disorders** |  |  |  |  |
| Tachycardia | 70 | 10 | - | 80 |
| Palpitations | 34 | 1 | - | 35 |
| Myocardial infarction | 22 | 5 | - | 27 |
| Cardiac disorder | 22 | 1 | - | 23 |
| Cardiac arrest | 22 | 1 | - | 23 |
| Cardiac failure | 20 | 1 | - | 21 |
| Bradycardia | 19 | 1 | 1 | 21 |
| Cardio-respiratory arrest | 18 | - | - | 18 |
| Arrhythmia | 13 | 1 | 1 | 15 |
| Angina pectoris | 11 | 1 | 1 | 13 |
| Cardiac failure acute | 8 | 2 | 1 | 11 |
| Acute myocardial infarction | 8 | - | - | 8 |
| Sinus tachycardia | 7 | 1 | - | 8 |
| Cardiovascular disorder | 7 | - | - | 7 |
| Ventricular extrasystoles | 4 | 2 | - | 6 |
| Sinus bradycardia | 6 | - | - | 6 |
| **Ear and labyrinth disorders** |  |  |  |  |
| Vertigo | 20 | 7 | - | 27 |
| Tinnitus | 13 | 4 | - | 17 |
| **Endocrine disorders** |  |  |  |  |
| Hyperprolactinaemia | 191 | 33 | 2 | 226 |
| Inappropriate antidiuretic hormone secretion | 7 | 1 | 1 | 9 |
| **Eye disorders** |  |  |  |  |
| Oculogyric crisis | 47 | 10 | - | 57 |
| Vision blurred | 39 | 6 | 1 | 46 |
| Visual impairment | 18 | 2 | 1 | 21 |
| Eye movement disorder | 16 | 4 | - | 20 |
| Dry eye | 9 | 3 | 1 | 13 |
| Gaze palsy | 8 | - | - | 8 |
| Accommodation disorder | 7 | - | - | 7 |
| Glaucoma | 6 | 1 | - | 7 |
| Blindness | 6 | - | - | 6 |
| Eyelid oedema | 5 | 1 | - | 6 |
| Photophobia | 4 | 2 | - | 6 |
| Diplopia | 4 | 2 | - | 6 |
| Eye pain | 5 | - | 1 | 6 |
| Lacrimation increased | 6 | - | - | 6 |
| Ocular hyperaemia | 6 | - | - | 6 |
| **Gastrointestinal disorders** |  |  |  |  |
| Nausea | 69 | 15 | 2 | 86 |
| Vomiting | 56 | 14 | - | 70 |
| Salivary hypersecretion | 56 | 7 | 3 | 66 |
| Dysphagia | 38 | 3 | 1 | 42 |
| Constipation | 36 | 1 | 1 | 38 |
| Diarrhoea | 35 | 2 | - | 37 |
| Abdominal pain | 22 | 4 | - | 26 |
| Dry mouth | 13 | 4 | 1 | 18 |
| Pancreatitis | 10 | 2 | - | 12 |
| Abdominal pain upper | 8 | 2 | 1 | 11 |
| Abdominal distension | 7 | - | 1 | 8 |
| Ileus | 8 | - | - | 8 |
| Toothache | 7 | - | - | 7 |
| Anal incontinence | 4 | 3 | - | 7 |
| Pancreatitis acute | 7 | - | - | 7 |
| Gastrointestinal disorder | 5 | 2 | - | 7 |
| Flatulence | 4 | 3 | - | 7 |
| Gastric disorder | 3 | 3 | - | 6 |
| Abdominal discomfort | 6 | - | - | 6 |
| **General disorders and administration site conditions** |  |  |  |  |
| Drug ineffective | 294 | 158 | 3 | 455 |
| Condition aggravated | 221 | 101 | 1 | 323 |
| Injection site pain | 151 | 36 | 3 | 190 |
| Fatigue | 130 | 48 | 2 | 180 |
| Malaise | 96 | 49 | 2 | 147 |
| Injection site induration | 101 | 32 | 1 | 134 |
| Treatment noncompliance | 109 | 17 | 4 | 130 |
| Death | 112 | 17 | - | 129 |
| Feeling abnormal | 78 | 26 | 1 | 105 |
| Injection site nodule | 70 | 19 | 2 | 91 |
| Pyrexia | 72 | 13 | 3 | 88 |
| Asthenia | 73 | 13 | - | 86 |
| Injection site mass | 49 | 12 | - | 61 |
| Therapeutic product effect decreased | 35 | 24 | 1 | 60 |
| Injection site swelling | 48 | 8 | 2 | 58 |
| Injection site erythema | 47 | 8 | - | 55 |
| Gait disturbance | 43 | 8 | 2 | 53 |
| General physical health deterioration | 41 | 5 | 2 | 48 |
| Sudden death | 44 | 2 | 1 | 47 |
| Injection site reaction | 44 | 3 | - | 47 |
| Therapeutic response decreased | 28 | 17 | - | 45 |
| Oedema peripheral | 36 | 5 | 1 | 42 |
| Pain | 34 | 6 | - | 40 |
| Peripheral swelling | 25 | 10 | 1 | 36 |
| Drug interaction | 34 | 1 | - | 35 |
| Chest pain | 23 | 4 | 1 | 28 |
| Oedema | 25 | 1 | - | 26 |
| Therapeutic product effect incomplete | 21 | 1 | 1 | 23 |
| Injection site inflammation | 20 | 2 | - | 22 |
| Hypothermia | 14 | 6 | - | 20 |
| Injection site cyst | 15 | 5 | - | 20 |
| Chills | 15 | 3 | 1 | 19 |
| Injection site oedema | 17 | 2 | - | 19 |
| Adverse event | 17 | 1 | - | 18 |
| Swelling | 13 | 4 | - | 17 |
| Adverse drug reaction | 14 | 2 | - | 16 |
| Induration | 14 | - | - | 14 |
| Hyperthermia | 12 | 2 | - | 14 |
| Symptom recurrence | 4 | 10 | - | 14 |
| Influenza like illness | 7 | 6 | - | 13 |
| Discomfort | 11 | 2 | - | 13 |
| Thirst | 11 | 2 | - | 13 |
| Face oedema | 9 | 2 | 2 | 13 |
| Chest discomfort | 13 | - | - | 13 |
| Injection site extravasation | 7 | 6 | - | 13 |
| Drug intolerance | 11 | 1 | - | 12 |
| Swelling face | 11 | 1 | - | 12 |
| Injection site warmth | 12 | - | - | 12 |
| Injection site pruritus | 10 | 1 | - | 11 |
| Drug ineffective for unapproved indication | 4 | 6 | 1 | 11 |
| Sluggishness | 9 | 2 | - | 11 |
| Inflammation | 9 | 1 | - | 10 |
| Unevaluable event | 4 | 5 | 1 | 10 |
| Gait inability | 9 | 1 | - | 10 |
| Illness | 9 | - | - | 9 |
| Drug resistance | 9 | - | - | 9 |
| Therapeutic response unexpected | 8 | 1 | - | 9 |
| Injection site granuloma | 9 | - | - | 9 |
| Application site erythema | 9 | - | - | 9 |
| Injection site rash | 6 | 1 | - | 7 |
| Nodule | 7 | - | - | 7 |
| Withdrawal syndrome | 6 | 1 | - | 7 |
| Injection site haemorrhage | 3 | 4 | - | 7 |
| Multiple organ dysfunction syndrome | 7 | - | - | 7 |
| Disease recurrence | 3 | 4 | - | 7 |
| Application site swelling | 6 | - | - | 6 |
| Adverse reaction | 5 | 1 | - | 6 |
| Feeling hot | 6 | - | - | 6 |
| Administration site swelling | 6 | - | - | 6 |
| **Hepatobiliary disorders** |  |  |  |  |
| Hepatic function abnormal | 12 | - | - | 12 |
| Liver disorder | 7 | - | - | 7 |
| Hepatic steatosis | 6 | - | 1 | 7 |
| Hepatic failure | 6 | - | - | 6 |
| **Immune system disorders** |  |  |  |  |
| Hypersensitivity | 37 | 2 | - | 39 |
| Anaphylactic reaction | 7 | - | - | 7 |
| Anaphylactic shock | 6 | - | - | 6 |
| **Infections and infestations** |  |  |  |  |
| Injection site abscess | 38 | 9 | 2 | 49 |
| Pneumonia | 36 | 5 | - | 41 |
| Urinary tract infection | 14 | 2 | - | 16 |
| Nasopharyngitis | 12 | - | 1 | 13 |
| Pneumonia aspiration | 12 | - | - | 12 |
| Respiratory tract infection | 11 | 1 | - | 12 |
| Abscess | 10 | 1 | 1 | 12 |
| Cellulitis | 6 | 3 | 1 | 10 |
| Infection | 7 | 2 | 1 | 10 |
| Sepsis | 9 | - | - | 9 |
| Influenza | 7 | 1 | - | 8 |
| Fungal infection | 7 | 1 | - | 8 |
| Injection site infection | 6 | 2 | - | 8 |
| Mastitis | 4 | 2 | - | 6 |
| Abscess limb | 6 | - | - | 6 |
| **Injury, poisoning and procedural complications** |  |  |  |  |
| Inappropriate schedule of product administration | 247 | 67 | 6 | 320 |
| Product use in unapproved indication | 193 | 76 | 4 | 273 |
| Incorrect dose administered | 133 | 20 | 1 | 154 |
| Product dose omission issue | 77 | 22 | 4 | 103 |
| Fall | 53 | 4 | 1 | 58 |
| Product use issue | 40 | 15 | - | 55 |
| Overdose | 47 | 2 | 1 | 50 |
| Wrong technique in product usage process | 30 | 19 | 1 | 50 |
| Underdose | 20 | 25 | 1 | 46 |
| Product administration error | 20 | 17 | 4 | 41 |
| Exposure during pregnancy | 31 | 4 | 1 | 36 |
| Incorrect route of product administration | 27 | 5 | - | 32 |
| Product administered at inappropriate site | 26 | 1 | - | 27 |
| Intentional overdose | 15 | 3 | - | 18 |
| Medication error | 13 | 3 | - | 16 |
| Road traffic accident | 13 | 2 | - | 15 |
| Foetal exposure during pregnancy | 12 | 2 | - | 14 |
| Accidental exposure to product | 12 | 2 | - | 14 |
| Intentional product use issue | 9 | 3 | 1 | 13 |
| Toxicity to various agents | 11 | 1 | - | 12 |
| Product prescribing error | 8 | 3 | 1 | 12 |
| Intentional product misuse | 8 | 2 | - | 10 |
| Prescribed overdose | 9 | 1 | - | 10 |
| Fracture | 9 | - | - | 9 |
| Product administered to patient of inappropriate age | 6 | 3 | - | 9 |
| Injury | 8 | 1 | - | 9 |
| Wrong product administered | 4 | 1 | 2 | 7 |
| Accidental overdose | 7 | - | - | 7 |
| Lack of injection site rotation | 5 | 2 | - | 7 |
| Contusion | 5 | - | 1 | 6 |
| **Investigations** |  |  |  |  |
| Weight increased | 313 | 73 | 13 | 399 |
| Blood prolactin increased | 210 | 25 | 4 | 239 |
| Weight decreased | 49 | 12 | 2 | 63 |
| Blood creatine phosphokinase increased | 50 | 5 | - | 55 |
| Electrocardiogram QT prolonged | 46 | 4 | - | 50 |
| Drug level decreased | 24 | 8 | - | 32 |
| Gamma-glutamyltransferase increased | 19 | 6 | - | 25 |
| Drug level increased | 17 | 3 | 1 | 21 |
| Transaminases increased | 17 | 3 | - | 20 |
| Heart rate increased | 13 | 6 | - | 19 |
| Hepatic enzyme increased | 15 | 3 | 1 | 19 |
| White blood cell count decreased | 15 | 3 | - | 18 |
| Blood pressure increased | 17 | 1 | - | 18 |
| Liver function test abnormal | 12 | 6 | - | 18 |
| Blood glucose increased | 15 | 1 | - | 16 |
| Platelet count decreased | 14 | 2 | - | 16 |
| Blood triglycerides increased | 12 | 3 | - | 15 |
| Aspartate aminotransferase increased | 12 | 2 | - | 14 |
| Alanine aminotransferase increased | 12 | 2 | - | 14 |
| Antipsychotic drug level below therapeutic | 8 | 4 | 1 | 13 |
| Blood cholesterol increased | 10 | 2 | - | 12 |
| Blood pressure decreased | 10 | 1 | 1 | 12 |
| Antipsychotic drug level increased | 9 | 2 | - | 11 |
| Neutrophil count decreased | 8 | 3 | - | 11 |
| Blood creatinine increased | 9 | 1 | - | 10 |
| Blood prolactin abnormal | 8 | 2 | - | 10 |
| C-reactive protein increased | 8 | 1 | - | 9 |
| Body temperature increased | 8 | - | - | 8 |
| White blood cell count increased | 7 | - | - | 7 |
| Semen volume decreased | 5 | 2 | - | 7 |
| Blood bilirubin increased | 7 | - | - | 7 |
| Hormone level abnormal | 4 | 2 | - | 6 |
| Blood testosterone decreased | 5 | 1 | - | 6 |
| Oxygen saturation decreased | 5 | 1 | - | 6 |
| **Metabolism and nutrition disorders** |  |  |  |  |
| Diabetes mellitus | 42 | 4 | - | 46 |
| Decreased appetite | 38 | 7 | - | 45 |
| Hyponatraemia | 25 | 6 | - | 31 |
| Increased appetite | 20 | 7 | 1 | 28 |
| Polydipsia | 19 | 4 | - | 23 |
| Obesity | 15 | 4 | - | 19 |
| Dehydration | 17 | 1 | - | 18 |
| Abnormal weight gain | 14 | 2 | 1 | 17 |
| Overweight | 13 | 3 | - | 16 |
| Type 2 diabetes mellitus | 14 | 1 | - | 15 |
| Hyperglycaemia | 11 | 4 | - | 15 |
| Hypokalaemia | 7 | 3 | - | 10 |
| Type 1 diabetes mellitus | 7 | 2 | - | 9 |
| Hypercholesterolaemia | 9 | - | - | 9 |
| Metabolic syndrome | 8 | - | - | 8 |
| Diabetic ketoacidosis | 8 | - | - | 8 |
| Hypoglycaemia | 6 | 1 | - | 7 |
| **Musculoskeletal and connective tissue disorders** |  |  |  |  |
| Musculoskeletal stiffness | 83 | 12 | 1 | 96 |
| Muscle rigidity | 64 | 6 | 2 | 72 |
| Rhabdomyolysis | 55 | 3 | - | 58 |
| Myalgia | 47 | 7 | 1 | 55 |
| Muscle spasms | 37 | 7 | 1 | 45 |
| Pain in extremity | 33 | 9 | 2 | 44 |
| Arthralgia | 25 | 8 | 1 | 34 |
| Back pain | 22 | 3 | 1 | 26 |
| Muscular weakness | 17 | 3 | - | 20 |
| Muscle twitching | 12 | - | - | 12 |
| Trismus | 11 | - | - | 11 |
| Mobility decreased | 7 | 2 | - | 9 |
| Muscle tightness | 6 | 3 | - | 9 |
| Limb discomfort | 4 | 2 | 1 | 7 |
| Muscle contracture | 6 | 1 | - | 7 |
| Bone pain | 6 | 1 | - | 7 |
| Neck pain | 7 | - | - | 7 |
| Joint stiffness | 6 | 1 | - | 7 |
| Muscle atrophy | 6 | - | - | 6 |
| **Neoplasms benign, malignant and unspecified (incl cysts and polyps)** |  |  |  |  |
| Pituitary tumour benign | 8 | 1 | - | 9 |
| Neoplasm malignant | 6 | 1 | - | 7 |
| Breast cancer | 6 | - | - | 6 |
| **Nervous system disorders** |  |  |  |  |
| Extrapyramidal disorder | 295 | 33 | 1 | 329 |
| Tremor | 135 | 28 | 5 | 168 |
| Akathisia | 139 | 19 | 1 | 159 |
| Headache | 105 | 22 | 2 | 129 |
| Somnolence | 89 | 25 | 5 | 119 |
| Dizziness | 96 | 16 | 4 | 116 |
| Neuroleptic malignant syndrome | 107 | 6 | - | 113 |
| Dyskinesia | 86 | 16 | - | 102 |
| Sedation | 72 | 23 | 1 | 96 |
| Parkinsonism | 81 | 7 | 2 | 90 |
| Dystonia | 60 | 7 | 1 | 68 |
| Tardive dyskinesia | 53 | 10 | 3 | 66 |
| Disturbance in attention | 42 | 7 | - | 49 |
| Speech disorder | 35 | 4 | 1 | 40 |
| Seizure | 37 | 2 | - | 39 |
| Memory impairment | 34 | 4 | 1 | 39 |
| Dysarthria | 31 | 2 | 1 | 34 |
| Balance disorder | 25 | 5 | 2 | 32 |
| Cognitive disorder | 26 | 3 | - | 29 |
| Epilepsy | 25 | 3 | - | 28 |
| Mental impairment | 25 | 2 | - | 27 |
| Hypokinesia | 25 | 2 | - | 27 |
| Paraesthesia | 18 | 6 | 1 | 25 |
| Loss of consciousness | 20 | 5 | - | 25 |
| Hypoaesthesia | 22 | 3 | - | 25 |
| Movement disorder | 20 | 2 | 1 | 23 |
| Syncope | 19 | 4 | - | 23 |
| Cerebrovascular accident | 17 | 6 | - | 23 |
| Hypersomnia | 16 | 5 | - | 21 |
| Altered state of consciousness | 20 | 1 | - | 21 |
| Lethargy | 18 | 2 | - | 20 |
| Sedation complication | 19 | - | - | 19 |
| Amnesia | 17 | 2 | - | 19 |
| Psychomotor hyperactivity | 16 | 2 | - | 18 |
| Coma | 15 | 1 | 1 | 17 |
| Bradykinesia | 14 | 1 | 1 | 16 |
| Hypertonia | 12 | 2 | - | 14 |
| Depressed level of consciousness | 12 | 2 | - | 14 |
| Drooling | 14 | - | - | 14 |
| Reduced facial expression | 10 | 3 | - | 13 |
| Restless legs syndrome | 10 | 2 | - | 12 |
| Migraine | 11 | - | 1 | 12 |
| Head discomfort | 10 | 2 | - | 12 |
| Motor dysfunction | 11 | - | - | 11 |
| Generalised tonic-clonic seizure | 9 | 2 | - | 11 |
| Parkinson’s disease | 11 | - | - | 11 |
| Aphasia | 9 | 1 | - | 10 |
| Electric shock sensation | 7 | 2 | - | 9 |
| Stupor | 9 | - | - | 9 |
| Sensory disturbance | 9 | - | - | 9 |
| Akinesia | 9 | - | - | 9 |
| Cerebral infarction | 9 | - | - | 9 |
| Burning sensation | 6 | 2 | - | 8 |
| Language disorder | 8 | - | - | 8 |
| Paralysis | 8 | - | - | 8 |
| Nervous system disorder | 5 | 2 | 1 | 8 |
| Cerebral haemorrhage | 7 | 1 | - | 8 |
| Cogwheel rigidity | 5 | 2 | - | 7 |
| Coordination abnormal | 6 | 1 | - | 7 |
| Ischaemic stroke | 5 | 1 | - | 6 |
| Facial paralysis | 6 | - | - | 6 |
| Psychomotor skills impaired | 4 | 2 | - | 6 |
| Taste disorder | 5 | 1 | - | 6 |
| Dysstasia | 6 | - | - | 6 |
| Status epilepticus | 6 | - | - | 6 |
| Incoherent | 5 | 1 | - | 6 |
| **Pregnancy, puerperium and perinatal conditions** |  |  |  |  |
| Abortion spontaneous | 5 | 3 | - | 8 |
| **Product issues** |  |  |  |  |
| Syringe issue | 13 | 6 | - | 19 |
| Product leakage | 7 | 2 | - | 9 |
| Device occlusion | 2 | 7 | - | 9 |
| Needle issue | 3 | 5 | - | 8 |
| Product complaint | 2 | 6 | - | 8 |
| Product colour issue | 5 | 1 | - | 6 |
| **Psychiatric disorders** |  |  |  |  |
| Schizophrenia | 324 | 174 | 6 | 504 |
| Psychotic disorder | 293 | 97 | 7 | 397 |
| Anxiety | 144 | 47 | 4 | 195 |
| Insomnia | 156 | 31 | 1 | 188 |
| Delusion | 89 | 54 | 1 | 144 |
| Psychotic symptom | 65 | 69 | 1 | 135 |
| Depression | 101 | 22 | 3 | 126 |
| Aggression | 99 | 21 | 1 | 121 |
| Hallucination | 89 | 25 | 1 | 115 |
| Hallucination, auditory | 83 | 29 | 2 | 114 |
| Restlessness | 91 | 17 | - | 108 |
| Agitation | 88 | 18 | - | 106 |
| Delirium | 77 | 27 | - | 104 |
| Completed suicide | 86 | 12 | - | 98 |
| Sleep disorder | 70 | 16 | 2 | 88 |
| Suicidal ideation | 78 | 10 | - | 88 |
| Psychiatric decompensation | 31 | 53 | - | 84 |
| Suicide attempt | 65 | 12 | - | 77 |
| Psychiatric symptom | 66 | 6 | 1 | 73 |
| Paranoia | 39 | 15 | 1 | 55 |
| Abnormal behaviour | 43 | 10 | 1 | 54 |
| Mania | 39 | 11 | - | 50 |
| Confusional state | 44 | 3 | 1 | 48 |
| Apathy | 39 | 8 | - | 47 |
| Libido decreased | 36 | 6 | 1 | 43 |
| Mental disorder | 32 | 10 | - | 42 |
| Irritability | 32 | 9 | - | 41 |
| Depressed mood | 26 | 12 | - | 38 |
| Schizoaffective disorder | 16 | 16 | - | 32 |
| Persecutory delusion | 16 | 13 | 1 | 30 |
| Catatonia | 24 | 4 | - | 28 |
| Thinking abnormal | 17 | 10 | - | 27 |
| Loss of libido | 25 | - | - | 25 |
| Drug abuse | 14 | 11 | - | 25 |
| Nervousness | 12 | 12 | - | 24 |
| Behaviour disorder | 16 | 5 | 1 | 22 |
| Social avoidant behaviour | 10 | 8 | 2 | 20 |
| Bipolar disorder | 8 | 10 | - | 18 |
| Intentional self-injury | 16 | 2 | - | 18 |
| Fear | 16 | 1 | - | 17 |
| Blunted affect | 12 | 4 | - | 16 |
| Euphoric mood | 14 | 2 | - | 16 |
| Stress | 9 | 5 | 1 | 15 |
| Hallucination, visual | 13 | 2 | - | 15 |
| Emotional poverty | 13 | 1 | - | 14 |
| Panic attack | 12 | 1 | - | 13 |
| Dissociation | 12 | 1 | - | 13 |
| Tension | 11 | 2 | - | 13 |
| Anhedonia | 10 | 3 | - | 13 |
| Enuresis | 12 | 1 | - | 13 |
| Eating disorder | 11 | 1 | - | 12 |
| Mood altered | 8 | 4 | - | 12 |
| Disorientation | 11 | 1 | - | 12 |
| Acute psychosis | 8 | 4 | - | 12 |
| Hypomania | 7 | 4 | - | 11 |
| Psychotic behaviour | 6 | 4 | - | 10 |
| Delusional disorder, unspecified type | 8 | 2 | - | 10 |
| Nightmare | 9 | 1 | - | 10 |
| Disinhibition | 9 | 1 | - | 10 |
| Emotional distress | 9 | - | - | 9 |
| Flat affect | 7 | 2 | - | 9 |
| Listless | 5 | 4 | - | 9 |
| Anger | 9 | - | - | 9 |
| Anorgasmia | 8 | 1 | - | 9 |
| Illusion | 7 | 2 | - | 9 |
| Mood swings | 7 | 2 | - | 9 |
| Bruxism | 7 | 1 | - | 8 |
| Affective disorder | 4 | 4 | - | 8 |
| Personality change | 8 | - | - | 8 |
| Obsessive thoughts | 5 | 2 | - | 7 |
| Major depression | 7 | - | - | 7 |
| Disturbance in social behaviour | 5 | 2 | - | 7 |
| Adjustment disorder with depressed mood | 6 | 1 | - | 7 |
| Emotional disorder | 6 | 1 | - | 7 |
| Libido disorder | 6 | 1 | - | 7 |
| Staring | 5 | 1 | - | 6 |
| Affect lability | 3 | 3 | - | 6 |
| Premature ejaculation | 4 | 2 | - | 6 |
| Drug dependence | 5 | 1 | - | 6 |
| Thought blocking | 4 | 2 | - | 6 |
| Tic | 3 | 2 | 1 | 6 |
| Psychomotor retardation | 6 | - | - | 6 |
| Impatience | 3 | 3 | - | 6 |
| **Renal and urinary disorders** |  |  |  |  |
| Urinary incontinence | 24 | 6 | - | 30 |
| Renal impairment | 15 | 4 | - | 19 |
| Urinary retention | 17 | 2 | - | 19 |
| Acute kidney injury | 13 | 4 | - | 17 |
| Dysuria | 14 | 1 | - | 15 |
| Renal failure | 8 | 3 | - | 11 |
| Incontinence | 4 | 3 | - | 7 |
| Pollakiuria | 5 | 2 | - | 7 |
| Polyuria | 4 | 1 | 1 | 6 |
| **Reproductive system and breast disorders** |  |  |  |  |
| Amenorrhoea | 132 | 14 | 2 | 148 |
| Galactorrhoea | 110 | 14 | 1 | 125 |
| Erectile dysfunction | 91 | 28 | - | 119 |
| Sexual dysfunction | 51 | 13 | 1 | 65 |
| Gynaecomastia | 44 | 7 | 1 | 52 |
| Priapism | 28 | 3 | - | 31 |
| Menstruation irregular | 23 | 1 | 1 | 25 |
| Ejaculation disorder | 13 | 7 | - | 20 |
| Ejaculation failure | 13 | 6 | - | 19 |
| Menstrual disorder | 15 | - | - | 15 |
| Breast pain | 9 | 3 | 1 | 13 |
| Breast discharge | 9 | - | - | 9 |
| Breast discomfort | 9 | - | - | 9 |
| Breast enlargement | 7 | 1 | 1 | 9 |
| Retrograde ejaculation | 8 | - | - | 8 |
| Infertility | 4 | 4 | - | 8 |
| **Respiratory, thoracic and mediastinal disorders** |  |  |  |  |
| Pulmonary embolism | 87 | 11 | 2 | 100 |
| Dyspnoea | 61 | 12 | 3 | 76 |
| Cough | 16 | - | 1 | 17 |
| Respiratory disorder | 14 | 2 | - | 16 |
| Epistaxis | 14 | 2 | - | 16 |
| Asphyxia | 14 | - | - | 14 |
| Respiratory failure | 14 | - | - | 14 |
| Aspiration | 13 | 1 | - | 14 |
| Choking | 7 | 1 | - | 8 |
| Chronic obstructive pulmonary disease | 8 | - | - | 8 |
| Lung disorder | 7 | - | - | 7 |
| Sleep apnoea syndrome | 5 | 2 | - | 7 |
| Nasal congestion | 5 | 1 | - | 6 |
| Pleural effusion | 6 | - | - | 6 |
| **Skin and subcutaneous tissue disorders** |  |  |  |  |
| Pruritus | 53 | 7 | 2 | 62 |
| Rash | 47 | 10 | - | 57 |
| Alopecia | 30 | 7 | 1 | 38 |
| Erythema | 34 | 1 | 1 | 36 |
| Hyperhidrosis | 30 | 6 | - | 36 |
| Eczema | 15 | 1 | - | 16 |
| Acne | 12 | 2 | - | 14 |
| Urticaria | 11 | 1 | - | 12 |
| Psoriasis | 8 | 3 | - | 11 |
| Angioedema | 9 | - | - | 9 |
| Skin reaction | 8 | - | - | 8 |
| Cold sweat | 5 | 1 | - | 6 |
| Skin discolouration | 5 | 1 | - | 6 |
| **Social circumstances** |  |  |  |  |
| Refusal of treatment by patient | 27 | 9 | 1 | 37 |
| Loss of personal independence in daily activities | 13 | 2 | - | 15 |
| Impaired work ability | 6 | 2 | - | 8 |
| Physical assault | 4 | 2 | - | 6 |
| **Surgical and medical procedures** |  |  |  |  |
| Hospitalisation | 96 | 13 | 4 | 113 |
| Self-medication | 7 | 3 | - | 10 |
| **Vascular disorders** |  |  |  |  |
| Hypotension | 44 | 8 | - | 52 |
| Hypertension | 44 | 5 | 1 | 50 |
| Deep vein thrombosis | 29 | 2 | 1 | 32 |
| Thrombosis | 13 | 1 | - | 14 |
| Pallor | 10 | 2 | - | 12 |
| Phlebitis | 8 | 4 | - | 12 |
| Orthostatic hypotension | 11 | 1 | - | 12 |
| Hot flush | 7 | - | - | 7 |
| Thrombophlebitis | 5 | 1 | - | 6 |
| Haemorrhage | 6 | - | - | 6 |
| Circulatory collapse | 4 | 2 | - | 6 |

**ADR reported in less then 5 cases in total have been excluded from this table*

**ESM table 3:** reporting odds ratios for PP-related ICSRs as compared to RG using both primary and secondary Standardized MedDRA queries.

| **SMQs** | **PP related ICSRs**  **N = 8056^b^** | **95% CI** | **ROR** |
| --- | --- | --- | --- |
| Psychosis and psychotic disorders | 1.331 | 0.85 - 0.99 | 0.92 |
| Medication errors | 642 | 0.92 - 1.14 | 1.03 |
| Lack of efficacy/effect | 640 | 0.97 - 1.19 | 1.07 |
| Sexual dysfunction | 287 | 1.23 - 1.7 | 1.45 |
| Suicide/self-injury | 267 | 0.58 - 0.78 | 0.67 |
| Hypersensitivity | 264 | 0.81 - 1.11 | 0.95 |
| Haemodynamic oedema. effusions and fluid overload | 229 | 1.18 - 1.7 | 1.42 |
| Gastrointestinal nonspecific symptoms and therapeutic procedures | 223 | 0.57 - 0.78 | 0.67 |
| Dyskinesia | 218 | 0.47 - 0.64 | 0.54 |
| Parkinson-like events | 207 | 1.06 - 1.53 | 1.27 |
| Depression (excl suicide and self injury) | 192 | 0.76 - 1.09 | 0.91 |
| Oropharyngeal conditions (excl neoplasms. infections and allergies) | 162 | 0.7 – 1.04 | 0.86 |
| Akathisia | 156 | 0.48 – 0.7 | 0.58 |
| Hostility/aggression | 143 | 0.41 – 0.61 | 0.50 |
| Dystonia | 140 | 0.61 – 0.91 | 0.75 |
| Embolic and thrombotic events. venous | 138 | 0.99 – 1.55 | 1.24 |
| Accidents and injuries | 127 | 0.54 - 0.83 | 0.67 |
| Hyperglycaemia/new onset diabetes mellitus | 115 | 0.34 - 0.51 | 0.42 |
| Liver related investigations, signs and symptoms | 113 | 0.72 - 1.15 | 0.91 |
| Noninfectious encephalopathy/delirium | 113 | 0.54 - 0.84 | 0.67 |
| Neuroleptic malignant syndrome | 110 | 0.71 - 1.15 | 0.91 |
| Ocular motility disorders | 98 | 0.73 - 1.21 | 0.94 |
| Haemorrhage terms (excl laboratory terms) | 91 | 0.6 - 1 | 0.77 |
| Haematopoietic leukopenia | 88 | 0.79 - 1.37 | 1.04 |
| Convulsions | 81 | 0.41 - 0.69 | 0.53 |
| Generalised convulsive seizures following immunisation | 79 | 0.42 - 0.71 | 0.55 |
| Conduction defects | 74 | 0.97 - 1.83 | 1.33 |
| Hypertension | 74 | 0.21 - 0.35 | 0.27 |
| Embolic and thrombotic events, vessel type unspecified and mixed arterial and venous | 71 | 0.41 - 0.71 | 0.54 |
| Angioedema | 70 | 0.55 - 0.97 | 0.73 |
| Drug abuse and dependence | 66 | 0.64 - 1.17 | 0.87 |
| Torsade de pointes, shock-associated conditions | 63 | 0.86 - 1.66 | 1.19 |
| Rhabdomyolysis/myopathy | 58 | 0.96 - 1.96 | 1.37 |
| Torsade de pointes/QT prolongation | 58 | 0.93 - 1.89 | 1.33 |
| Embolic and thrombotic events, arterial | 52 | 0.41 - 0.77 | 0.56 |
| Ischaemic central nervous system vascular conditions | 51 | 0.44 - 0.84 | 0.60 |
| Shock-associated circulatory or cardiac conditions (excl torsade de pointes) | 51 | 0.61 - 1.21 | 0.86 |
| Pregnancy, labour and delivery complications and risk factors (excl abortions and stillbirth) | 50 | 0.39 - 0.75 | 0.54 |
| Acute renal failure | 48 | 0.68 - 1.41 | 0.98 |
| Dyslipidaemia | 48 | 0.42 - 0.82 | 0.59 |
| Immune-mediated/autoimmune disorders | 44 | 0.79 - 1.73 | 1.17 |
| Infective pneumonia | 44 | 0.51 - 1.05 | 0.73 |
| Haematopoietic thrombocytopenia | 42 | 0.77 - 1.73 | 1.15 |
| Haemorrhagic central nervous system vascular conditions | 42 | 0.47 - 0.97 | 0.67 |
| Non-haematological malignant tumours | 42 | 0.51 - 1.07 | 0.74 |
| Hyponatraemia/SIADH | 40 | 0.64 - 1.43 | 0.96 |
| Cardiac failure | 38 | 0.61 - 1.36 | 0.91 |
| Hepatic failure, fibrosis and cirrhosis and other liver damage-related conditions | 38 | 0.57 - 1.26 | 0.84 |
| Noninfectious diarrhoea | 37 | 0.52 - 1.15 | 0.78 |
| Myocardial infarction | 36 | 0.38 - 0.83 | 0.56 |
| Fertility disorders | 32 | 1.51 - 4.8 | 2.69 |
| Respiratory failure | 29 | 0.4 - 0.95 | 0.62 |
| Hearing impairment | 28 | 0.75 - 2.05 | 1.24 |
| Vestibular disorders | 28 | 0.23 - 0.52 | 0.35 |
| Periorbital and eyelid disorders | 26 | 0.69 - 1.93 | 1.16 |
| Acute central respiratory depression | 25 | 0.38 - 0.97 | 0.61 |
| Other ischaemic heart disease | 23 | 0.62 - 1.79 | 1.05 |
| Anaphylactic reaction | 22 | 0.53 - 1.52 | 0.90 |
| Anaphylactic/anaphylactoid shock conditions | 22 | 0.53 - 1.52 | 0.90 |
| Peripheral neuropathy | 22 | 0.66 - 2 | 1.15 |
| Conjunctival disorders | 20 | 0.85 - 2.98 | 1.59 |
| Gastrointestinal obstruction | 20 | 0.89 - 3.18 | 1.68 |
| Non-haematological tumours of unspecified malignancy | 19 | 0.97 - 3.77 | 1.92 |
| Acute pancreatitis | 18 | 0.42 - 1.29 | 0.73 |
| Agranulocytosis | 18 | 0.54 - 1.76 | 0.97 |
| Cardiac arrhythmia terms. nonspecific | 18 | 0.29 - 0.86 | 0.50 |
| Dehydration | 18 | 0.52 - 1.69 | 0.94 |
| Lacrimal disorders | 18 | 0.61 - 2.09 | 1.13 |
| Supraventricular tachyarrhythmias | 18 | 0.24 - 0.68 | 0.40 |
| Congenital, familial and genetic disorders | 17 | 0.62 - 2.2 | 1.17 |
| Hepatitis, non-infectious | 16 | 0.68 - 2.64 | 1.34 |
| Taste and smell disorders | 16 | 0.53 - 1.9 | 1.01 |
| Chronic kidney disease | 15 | 0.28 - 0.9 | 0.50 |
| Haematopoietic cytopenias affecting more than one type of blood cell | 15 | 0.78 - 3.36 | 1.62 |
| Oropharyngeal allergic conditions | 15 | 0.32 - 1.05 | 0.58 |
| Extravasation events (injections, infusions and implants) | 14 | 0.53 - 2.09 | 1.06 |
| Severe cutaneous adverse reactions | 14 | 0.76 - 3.47 | 1.63 |
| Cholestasis and jaundice of hepatic origin | 13 | 0.32 - 1.17 | 0.61 |
| Glaucoma | 13 | 0.75 - 3.59 | 1.64 |
| Sepsis | 13 | 0.47 - 1.87 | 0.94 |
| Biliary system related investigations, signs and symptoms | 12 | 0.34 - 1.33 | 0.67 |
| Toxic-septic shock conditions | 12 | 0.41 - 1.67 | 0.82 |
| Ventricular tachyarrhythmias | 12 | 0.28 - 1.06 | 0.55 |
| Hypokalaemia | 11 | 0.4 - 1.73 | 0.83 |
| Cardiomyopathy | 9 | 0.38 - 1.92 | 0.85 |
| Gastrointestinal nonspecific dysfunction | 9 | 0.25 - 1.16 | 0.54 |
| Hypoglycaemic and neurogenic shock conditions | 9 | 0.36 - 1.79 | 0.80 |
| Hypovolaemic shock conditions | 9 | 0.31 - 1.49 | 0.68 |
| Neonatal disorders | 9 | 0.45 - 2.45 | 1.05 |
| Termination of pregnancy and risk of abortion | 9 | 0.15 - 0.65 | 0.32 |
| Biliary tract disorders | 8 | 0.2 - 0.95 | 0.43 |
| Breast malignant tumours | 8 | 0.2 - 0.98 | 0.45 |
| COVID-19 | 8 | 0.63 - 4.76 | 1.73 |
| Disorders of sinus node function | 8 | 0.48 - 3.06 | 1.21 |
| Foetal disorders | 8 | 0.7 - 5.81 | 2.02 |
| Gastrointestinal haemorrhage | 8 | 0.17 - 0.82 | 0.38 |
| Hypoglycaemia | 8 | 0.15 - 0.7 | 0.33 |
| Tendinopathies and ligament disorders | 8 | 0.23 - 1.12 | 0.50 |
| Functional lactation disorders | 7 | 0.29 - 1.73 | 0.70 |
| Gastrointestinal nonspecific inflammation | 7 | 0.35 - 2.24 | 0.88 |
| Gastrointestinal ulceration | 7 | 0.22 - 1.25 | 0.53 |
| Noninfectious meningitis | 7 | 0.48 - 3.65 | 1.32 |
| Asthma/bronchospasm | 6 | 0.3 - 2.23 | 0.82 |
| Demyelination | 6 | 0.3 - 2.23 | 0.82 |
| Haematological malignant tumours | 6 | 0.36 - 2.83 | 1.01 |
| Lens disorders | 6 | 0.33 - 2.49 | 0.91 |
| Oropharyngeal infections | 6 | 0.23 - 1.56 | 0.60 |
| Retinal disorders | 6 | 0.26 - 1.83 | 0.70 |
| Tachyarrhythmia terms. nonspecific | 6 | 0.33 - 2.49 | 0.91 |
| Thrombophlebitis | 6 | 0.36 - 2.83 | 1.01 |
| Vasculitis | 6 | 0.76 - 12.09 | 3.02 |
| Haematopoietic erythropenia | 5 | 0.15 - 1.06 | 0.40 |
| Malignant lymphomas | 5 | 0.38 - 4.13 | 1.26 |
| Gastrointestinal premalignant disorders | 4 | 0.25 - 2.95 | 0.86 |
| Hyperthyroidism | 4 | 0.32 - 4.5 | 1.21 |
| Hypothyroidism | 4 | 0.12 - 1.06 | 0.36 |
| Ischaemic colitis | 4 | 0.32 - 4.5 | 1.21 |
| Liver infections | 4 | 0.14 - 1.31 | 0.43 |
| Noninfectious myocarditis/pericarditis | 4 | 0.32 - 4.5 | 1.21 |
| Ocular infections | 4 | 0.55 - 16.51 | 3.02 |
| Osteoporosis/osteopenia | 4 | 0.23 - 2.51 | 0.76 |
| Arrhythmia related investigations, signs and symptoms | 3 | 0.17 - 2.5 | 0.65 |
| Arthritis | 3 | 0.06 - 0.69 | 0.21 |
| Conditions associated with central nervous system haemorrhages and cerebrovascular accidents | 3 | 0.06 - 0.69 | 0.21 |
| Dementia | 3 | 0.06 - 0.66 | 0.20 |
| Gallbladder related disorders | 3 | 0.11 - 1.48 | 0.41 |
| Gastrointestinal perforation | 3 | 0.11 - 1.34 | 0.38 |
| Gingival disorders | 3 | 0.11 - 1.34 | 0.38 |
| Lipodystrophy | 3 | 0.3 - 7.49 | 1.51 |
| Opportunistic infections | 3 | 0.17 - 2.5 | 0.65 |
| Uterine and fallopian tube malignant tumours | 3 | 0.3 - 7.49 | 1.51 |

COVID-19: Coronavirus disease; ICSRs: Individual Case Safety Reports; PP: paliperidone palmitate; SIADH: syndrome of inappropriate antidiuretic hormone secretion. ^a^ICSRs distribution by SMQ is not mutually exclusive
